# Mechanisms of flavonoids in quinoa’s response to flooding stress in grain filling stage

**DOI:** 10.3389/fpls.2025.1565697

**Published:** 2025-05-21

**Authors:** Guofei Jiang, Xuqin Wang, Qingwen Jiang, Yutao Bai, Lingyuan Zhang, Ping Zhang, Junna Liu, Li Li, Hanxue Li, Liubin Huang, Shan Zhang, Peng Qin

**Affiliations:** College of Agronomy and Biotechnology, Yunnan Agricultural University, Kunming, China

**Keywords:** quinoa, flooding stress, flavonoids, metabolome, transcriptome

## Abstract

Quinoa is a new crop with high nutritional value. Flooding stress plays an important role in constraining the growth and development of quinoa during the filling stage, and flavonoids have been shown to have important roles in abiotic plant stress; however, the mechanisms by which flavonoids respond to quinoa flooding stress during the filling stage are not clear. Therefore, we used Dian quinoa-1844 as the material and employed transcriptomics, metabolomics and bioinformatics techniques to study quinoa leaves under flooding stress during the filling stage. The results showed that 433 flavonoid metabolites were detected in the metabolome. Genes related to flavonoids in the transcriptome were significantly enriched in both GO and KEGG. Integrated transcriptomic and metabolomic analyses revealed 18 flavonoid metabolites and 30 genes exhibiting significant alterations under stress treatment. These 30 genes regulate flavonoid accumulation by modulating the activity of enzymes such as F3H, CHI, and CHS, thereby enhancing quinoa’s resistance to flooding stress. Network interaction analysis identified 5 core transcription factors, 2 core structural genes, and 4 key metabolites. These components synergistically regulate flavonoid biosynthesis to alleviate oxidative damage caused by flooding. This study elucidated the roles and mechanisms of flavonoids in quinoa’s response to flooding stress, providing a theoretical basis for selecting and breeding quinoa varieties with high flooding tolerance.

## 1 Introduction

Quinoa (*Chenopodium quinoa* Willd.) is a dicot herb of the amaranth family (Amaranthaceae) and *Chenopodium* genus, originating from the Andes Mountains (García-Parra et al., 2020; Vilcacundo and Hernández-Ledesma, 2017). It is recognized by the United Nations International Food and Agriculture Organization (FAO) as the only crop-plant that can meet the basic nutritional needs of the human body. It enjoys the reputation of “golden grain” (Angeli et al., 2020). Quinoa is rich in flavonoids, proteins, amino acids, minerals, polyphenols, vitamins, dietary fiber, unsaturated fatty acids, and many other components (Abderrahim et al., 2015; Lamothe et al., 2015; Tang et al., 2015). Many studies have shown that the protein, phenolic acids, flavonoids, saponins and other substances in quinoa are important in promoting human health by lowering blood lipids, antidiabetic, and antioxidant (Cao et al., 2020; Mudgil et al., 2020; Daliri et al., 2021).

The grain-filling stage, the final and most critical phase of cereal growth, directly determines grain weight and final yield. However, this process is highly vulnerable to diverse abiotic stresses (Teng et al., 2023). Flavonoid content in plants has been shown to correlate strongly with stress resistance (Zhuang et al., 2023; Meng et al., 2021). Therefore, investigating the synthesis mechanisms of flavonoids in quinoa during the grain-filling stage under flooding stress holds significant scientific and agronomic importance. Quinoa has been extensively cultivated in the Yunnan, Gansu, and Qinghai provinces of China. In these regions, the optimal sowing period consistently occurs in mid-May, with the critical filling stage spanning July to August. Notably, this phenological phase coincides with rainy summer across all three provinces (Wang and LinHo, 2002). Excessive precipitation during this period induces elevated soil water retention capacity, thereby subjecting quinoa to flooding stress (Guo et al., 2024). While adequate water availability is essential for normal plant growth, the resultant flooding has emerged as one of the predominant abiotic stressors impeding quinoa cultivation in China. During flooding stress, plant root respiration is inhibited and toxic substances accumulate, affecting nutrient growth and detrimental to reproductive growth, ultimately leading to yield loss or even plant death (Zhou et al., 2020). Plants adapt to inundation stress by osmoregulation and osmoprotection to maintain normal life activities. Most plant species cannot survive prolonged inundation or soil waterlogging. Previous studies have been reported on the mechanism of rice tolerance to flooding (Loreti et al., 2016), where more cellular gaps and better structural integration of chloroplast membranes were observed in the leaves of flood-tolerant varieties, probably due to increased ethylene content, decreased ABA content and reduced O^2-^ accumulation (Luan et al., 2018). The use of exogenous ethylene may increase leaf photosynthesis by promoting stomatal opening under waterlogging stress (Wang et al., 2016). Currently, global warming and erratic precipitation have serious impacts on crops, and the study of flooding tolerance mechanisms in quinoa is important for improving quinoa yield as well as effectively responding to climate change.

Flavonoids are water-soluble secondary metabolites that are widely present in plants and mainly include six classes of compounds: chalcones, flavones, flavonols, flavanediols, anthocyanin glycosides, and condensed tannins (Winkel-Shirley, 2001b). Flavonoids play an important role in plant growth and development and have a variety of biological functions for the plant body itself, participating in a range of physiological processes including UV protection, pollination, seed development, pigmentation and chemosensitivity (Winkel-Shirley, 2001a, 2001). Flavonoids also play an important role in plant abiotic stress resistance due to their strong antioxidant capacity to scavenge excess reactive oxygen species (ROS) induced in plants by various abiotic stresses. Studies have shown that the stress resistance of numerous plants is closely related to the flavonoid content in the body (Zhuang et al., 2023; Meng et al., 2021). Overexpression of key genes involved in flavonoid biosyntheses such as chalcone isomerase 2 (*CHI2*), *F3H*, and production of anthocyanin pigment 1 (*PAP1*) increases flavonoid content in plants such as arabidopsis, rice and pohlia nutans, among others, and increase the flavonoid content and antioxidant properties, thereby significantly enhancing the resistance of these plants to salt, drought and cold damage (Nakabayashi et al., 2014; Jayaraman et al., 2021; Liu et al., 2022a). Salt stress significantly modulates the flavonoid biosynthesis pathway in germinating kidney beans and activates key enzymes such as chalcone isomerase (CHI) and flavonol synthase (FLS), demonstrating the critical role of flavonoid metabolism in salt stress adaptation (Zhang et al., 2022). Integrated multi-omics analysis in maize under lead stress revealed that DEMs and DEGs were co-enriched in the flavonoid biosynthesis pathway, with 20 flavonoid-related genes identified, further validating the pivotal function of this pathway in heavy metal stress responses (Han et al., 2024). Furthermore, soybean seeds with elevated flavonoid levels exhibit enhanced tolerance to herbicides, oxidative stress, and saline-alkaline stress during germination, highlighting their multi-stress synergistic defense capacity (Jiang et al., 2024). In drought-stressed Morus alba var. pendula, PEG-6000 treatment not only significantly increased flavonoid accumulation but also upregulated the expression of phenylpropanoid biosynthesis genes (Chen et al., 2024). Therefore, the outstanding antioxidant capacity of flavonoids plays an important role in mitigating oxidative damage and can effectively improve plant tolerance to various abiotic stresses (Singh et al., 2021). However, the response mechanisms of flavonoid compounds to flooding stress during the filling stage of quinoa remain uninvestigated to date. The findings of this study will address this critical knowledge gap.

In recent years, studies combining transcriptome and metabolome associations to analyze plant flavonoids in response to flooding stress have been rare. To explore this issue in depth, this experiment was conducted to comprehensively analyze the dynamic changes of leaf flavonoid metabolites in quinoa during the filling stage under flooding stress using an independently selected high-generation quinoa line as the material, and ultra-high-performance liquid chromatography and tandem mass spectrometry (UPLC-MS/MS) technology. At the same time, differentially expressed genes related to quinoa flooding stress were screened out with the help of transcriptomics, thus providing a theoretical basis for the study of the molecular mechanism of flavonoids in response to quinoa flooding stress as well as the selection and breeding of quinoa flooding-tolerant varieties.

## 2 Materials and methods

### 2.1 Test materials and treatments

This experiment selected Dianli-1844, a high generation red quinoa strain independently selected by Yunnan Agricultural University, as the experimental material. During the planting process, this strain is extremely sensitive to flooding stress during the filling stage. After encountering stress, the plants quickly wilt and the leaves significantly turn yellow, so it was selected as the research object. The material is planted in the Modern Agricultural Education and Research Base of Yunnan Agricultural University in Xundian County, Kunming City (E 102°41′, N 25°20′, Kunming, China). Uniform-sized seeds were selected and sown using hill-drop sowing method in 50 cm × 50 cm × 50 cm pots. Each flowerpot is equipped with 6 sowing holes, with 5 seeds per hole and a plant spacing of 20cm. Thinning was implemented when seedlings reached 5 cm height (corresponding to the emergence of the fourth true leaf), retaining only one robust quinoa plant per hole post-thinning. The first stage is managed according to normal cultivation management techniques, during the growth period, the average temperature was 25.6°C, and the sunshine duration was approximately 12 h. Flooding stress was simulated during the quinoa filling stage (15 days after flowering) using a double-pot system. Artificial flooding was implemented by injecting water into the pots to maintain a flooding depth 10 cm above the soil surface, with daily water replenishment to compensate for evapotranspiration losses. On day 7 post-flooding, when leaves exhibited significant chlorosis compared to the control group, the first sampling was conducted. Following this, the outer pot was removed to initiate gravitational drainage. The second sampling occurred 7 days after drainage completion, during which the sixth fully expanded leaf from the apex was excised, flash-frozen in liquid nitrogen, and subsequently stored at −80°C for downstream analyses. Sampled leaves were analyzed for metabolome and transcriptome (Wuhan Metware Biotechnology Co. Ltd., (www.metware), 3 biological replicates, a total of 12 samples. Specifically, three distinct quinoa plants were selected as biological replicates, and leaves were collected from identical developmental positions on each plant. These harvested leaves were divided into three aliquots for transcriptomic sequencing, metabolomic profiling, and other parameter measurements. In the analyses herein, TR represents 7 days of flooding treatment, R represents 7 days of drainage recovery, and C1 and C2 represent 7 and 14 days of normal growth control, respectively.

### 2.2 Flavonoid content assay

Flavonoid content was determined using a kit purchased from Wuhan Puyinte Bioengineering Co., Ltd, Wuhan, China (http://www.pytbio.com), and the assay process strictly followed the manufacturer’s instructions for experiments and calculations.

### 2.3 Flavonoid metabolite extraction detection

The quinoa leaf samples under different treatments were placed in a lyophilizer (Scientz-100F) and freeze-dried under vacuum, ground to powder form using a grinder (MM400, Retsch), dissolved in methanol extract, and the supernatant was taken by vortex centrifugation and filtered for UPLC-MS/MS analysis. The data acquisition instrumentation system mainly consisted of Ultra Performance Liquid Chromatography (UPLC) (SHIMADZU Nexera X2, https://www.shimadzu.com.cn/) and Tandem mass spectrometry (MS/MS) (Applied Biosystems 4500 QTRAP, http://www.appliedbiosystems.com.cn/). Based on the database (MWDB; http://en.metware.cn/list/27.html, accessed on 15 June 2021), substance characterization by secondary mass spectrometry interest and metabolite quantification by multiple reaction monitoring (MRM) modes using triple quadrupole mass spectrometry, metabolite profiling data were obtained for different samples, the Peak area integration was performed and the integral correction was applied (Kogenaru et al., 2012). Quality Control (QC) samples were prepared from a mixture of quinoa plant sample extracts to analyze the reproducibility of the samples under the same treatments.

### 2.4 Transcriptome sequencing and data analysis

Transcriptome sequencing was performed by Beijing NoZvogene Technology Co., Ltd. (www.noZvogene.com.cn), including RNA extraction and detection, library construction and quality inspection, and machine sequencing. Before library construction, the integrity of RNA was first judged by agarose gel electrophoresis, and then Qubit 2.0 fluorometer and Agilent 2100 bioanalyzer were used to detect the concentration and integrity of RNA to ensure that the quality of RNA was qualified. Library construction was performed using Illumina’s NEBNext^®^ UltraTM RNA Library Prep Kit. After the library construction was completed, the Qubit2.0 Fluorometer was used for preliminary quantification, and the library was diluted to 1.5 ng/ul. Subsequently, the insert size of the library was detected using Agilent 2100 bioanalyzer, and after the insert size was the expected one, the effective concentration of the library was quantified (the effective concentration of the library was higher than 2 nM) by RT-qPCR to ensure the quality of the library. After passing the library check, different libraries were first pooled according to the effective concentration and target downstream data volume, and then sequenced using the Illumina platform, and 150bp paired-end reads were generated. The downlinked data were filtered using fastp v 0.19.3, and the filtering criteria were as follows: firstly, reads with adapters were removed; when the N content of any sequencing reads exceeded 10% of the number of bases of the reads, the paired reads were removed; when the number of low-quality (Q ≤ 20) bases in any sequencing reads exceeded 50% of the number of bases of the reads, the paired reads were removed, and the clean reads were obtained; the index was constructed using HISAT v2.1.0, and the clean reads were compared with the specified reference data, and the indexes were constructed using HISAT v2.1.0. reads, remove these paired reads when the number of bases in any one of the sequenced reads exceeds 50% of the number of bases in the reads, get clean reads, use HISAT v2.1.0 to construct indexes, and sequence the clean reads against the specified reference genome (GCF001683475.1ASM168347v1_genomic. fna. gz), and get the Mapped Data, and use StringTie v1.3.4d to perform new gene-prediction using the featureCounts v1.6.2 for gene comparison, and then calculate Fragments Per Kilobase of exon model per Million mapped fragments (FPKM) for each gene based on gene length. Differential expression analysis between two groups was performed using DESeq2 v1.22.1, and the Benjamini-Hochberg method was used to correct for P-values, resulting in a False Discovery Rate (FDR). The screening criteria for differentially expressed genes are | log2Fold Change |>=1 and FDR<0.05. Functional annotation of differentially expressed genes was performed using KEGG, GO (Gene Ontology), KOG (Karyotic Orthologous Groups), PfAM, Swiss-Prot, TrEMBL, and NR databases. Pathway significance enrichment analysis was performed as a hypergeometric distribution test in terms of pathways in the KEGG database to identify Pathways that were significantly enriched in differentially expressed genes compared to the whole genomic background.

### 2.5 Combined transcriptome and metabolome analysis

To better understand the relationship between genes and metabolites, differential genes and differential metabolites under the same grouping were simultaneously mapped to KEGG pathway maps based on the results of differential metabolite and differential gene analyses, and bar charts and bubble charts were plotted based on the results of the enrichment analyses, demonstrating the degree of differences in the enrichment of metabolites and gene pathways. The genes and metabolites detected in each treatment were correlated, and the Pearson correlation coefficients of the genes and metabolites were calculated using the COR program in R. Differential genes and differential metabolites were correlated, and the results with Pearson correlation coefficients greater than 0.8 were selected and the correlation coefficients were plotted in a clustered heatmap (Chong and Xia, 2018). Canonical correlation analysis (CCA) selected all the differential genes and differential metabolites to establish the O2PLS model and preliminarily judged the variables with higher correlation and weight in different data sets through the loading diagram (Bouhaddani et al., 2016).

### 2.6 Transcription factor identification analysis and RT-qPCR

Transcription factor families were identified and annotated using ITAK (IAITAM, Canton, OH, USA) software (Perez-Rodriguez et al., 2010; Jin et al., 2014). The core genes were subjected to protein sequence extraction at the NCBI website, and blasts were selected from the plantTFDB database (Jin et al., 2014) for transcription factor analysis prediction and further understanding of the functions of the core genes. RNA was extracted from leaves of the Dianli-1844 quinoa strain under various treatments (C1, C2, TR, R) for RT-qPCR analysis. The materials used for RT-qPCR and RNA-seq analysis were derived from the same batch of experimental plants. Each reaction was repeated three times with primer design using BeaconDesign 7.9, the internal reference gene was TUB-6 (**Supplementary Table 1**). The reagents were PerfectStartTM SYBR qPCR Supermix (TransGen Biotech, Beijing, China). The ABI Prism 7500 instrument (Applied Biosystems, USA) was utilized, and the 2^-ΔΔCт^ method was applied to analyze the normalized expression of each sample (Livak and Schmittgen, 2001).

### 2.7 Data statistics and analysis

Physiological and morphological indicators and RT qPCR data were processed and statistically analyzed using Excel 2019 (Microsoft Corporation, Redmond, Washington, WA, USA) and SPSS 26.0 for Windows (IBM Corp., Armonk, NY, USA). Significant differences were identified using a two-way analysis of variance (ANOVA). Results with P<0.05 were deemed statistically significant.

Qualitative and quantitative analysis steps of flavonoid metabolites: During instrumental analysis, one quality control sample was inserted for every 10 samples analyzed by the assay to monitor the reproducibility of the analytical process. Multivariate statistical analysis was used to maximize the preservation of the original information, and the data were simplified and downscaled to create a reliable mathematical model, and the built-in statistical prcomp function (Fraga et al., 2010) of the R software (www.r-project.org/) was used to show the analyses between the groups and the differences between the sample groups. Heatmap was drawn by R software heatmap for hierarchical cluster analysis, and Orthogonal Projections to Latent Structures - Discriminant Analysis (OPLS-DA) was utilized for differential variable screening (Chen et al., 2009; Eriksson et al., 2005), furthermore, Variable Importance in Projection (VIP) was obtained for multivariate analysis of OPLS-DA model and combined with Fold Change to further screen differential metabolites (Thevenot et al., 2015). Significantly regulated metabolites between groups were further analyzed by screening differential metabolites with VIP ≥ 1, fold change ≥ 2 and fold change ≤ 0.5. If the difference in metabolites between the control group and the experimental group is more than 2 times or less than 0.5, it is considered significant. The identified metabolites were annotated through the Kyoto Encyclopedia of Genes and Genomes (KEGG) Compound Database (http://www.kegg.jp/kegg/compound/) and then the annotated metabolites were mapped to the KEGG Pathway Database (http://www.kegg.jp/kegg/pathway.html) (Thevenot et al., 2015). Pathways with significantly regulated metabolites were entered into MSEA (metabolite sets enrichment analysis) and their significance was determined by the p-value of the hypergeometric test.

## 3 Results and analysis

### 3.1 Changes in the phenotype of quinoa and the total flavonoid content in its leaves after flooding and drainage treatments

**Figure 1:**
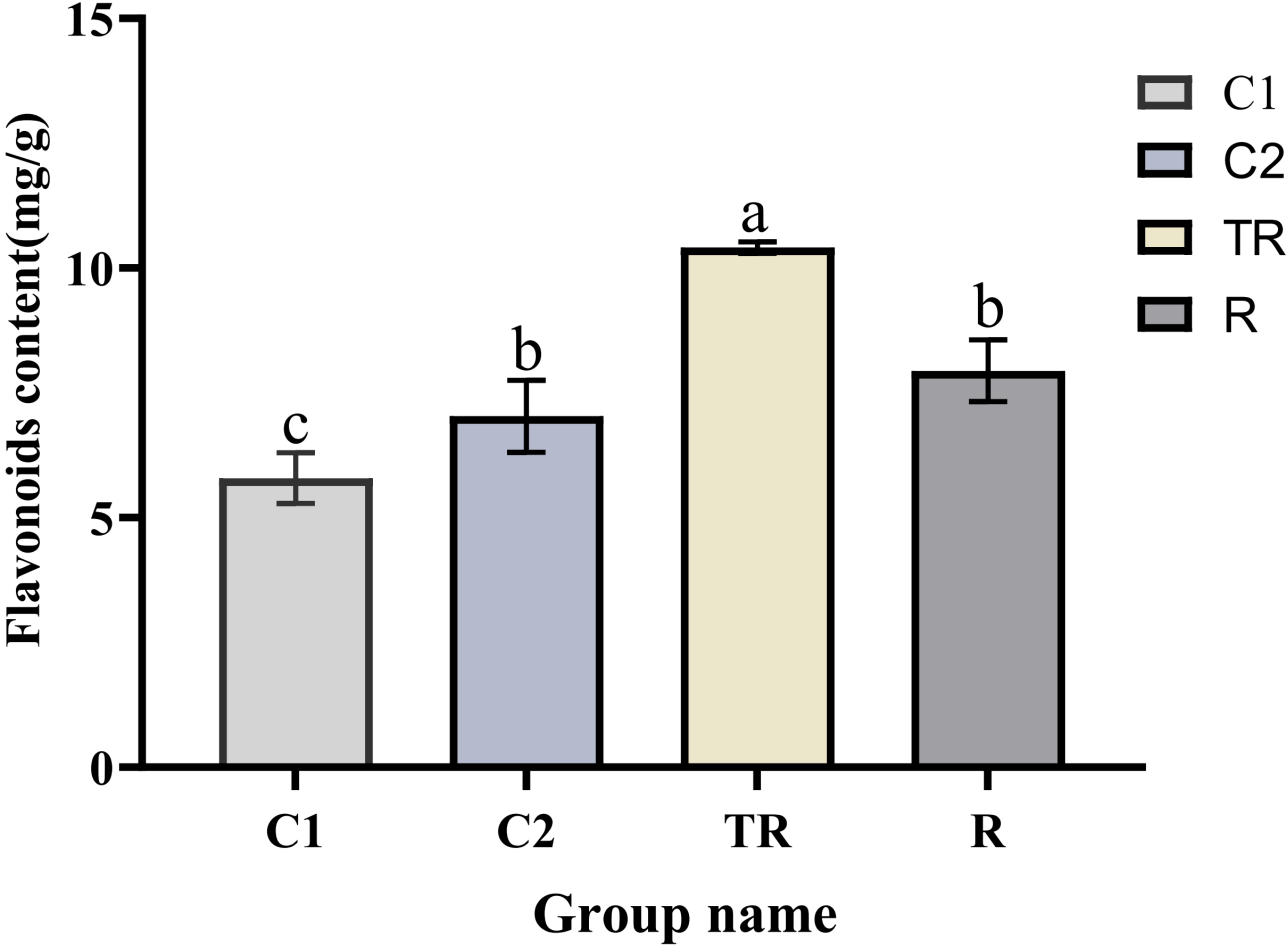
Changes in flavonoid content in quinoa leaves under flooding and recovery treatments. Different letters (a-c) indicate significant differences (*P* < 0.05) and the same letter indicates no significance (*P* > 0.05).

Following flooding and post-drainage recovery treatments, quinoa exhibited significant phenotypic alterations, indicating high sensitivity of its growth and developmental processes to flooding stress (**Supplementary Table 2**). Under flooding stress, the dry weight, fresh weight, and leaf area of quinoa leaves were significantly reduced. Following post-drainage recovery, plant height, leaf area, and dry leaf weight remained markedly lower compared to the control group. These results indicate that flooding stress inflicted irreversible damage on quinoa, leading to persistently impaired growth and development even after recovery. The total flavonoid content of quinoa leaves showed significant changes under flooding stress and recovery treatment (**Figure 1**). Overall, the flavonoid content of leaves under both flooding and recovery treatments was higher than that of the control. The TR flavonoid content was the highest and significantly higher than that of the other treatments, and the content decreased after drainage treatment. It indicated that flavonoids played a role in quinoa coping with flooding stress.

### 3.2 Qualitative and quantitative analysis of flavonoid metabolites

We constructed principal component analysis score plots (**Supplementary Figure 1A**) with flavonoids to understand the classification of flavonoids in the 12 samples. As shown in the figure, the QC samples have a high degree of overlap, indicating good instrumental stability and data reliability. The 12 samples showed good biological reproducibility within groups and good differentiation between groups, reaching 39.34% for PC1 and 71.03% overall, indicating that the flavonoids were reproducible in the 12 samples and reliable for subsequent analysis. Qualitative and quantitative analyses were conducted on flavonoids in quinoa leaves, with a total of 433 flavonoid metabolites detected (**Supplementary Table 3**). Of which there were 16 chalcones, 20 flavanols, 44 flavanones, 10 flavanonols, 150 flavones, 158 flavonols, 8 isoflavones, and 27 other flavonoids (**Supplementary Figure 1B**). Flavones and flavonols had the highest numbers, 150 and 158 respectively, while isoflavones and flavanonols had the fewest, only 8 and 10 respectively. Clustering heatmap analysis of 433 flavonoid metabolites (**Supplementary Figure 1C**) revealed a distinct separation between the up- and down-regulated clusters in the TR group compared to C1, indicating marked alterations in flavonoid accumulation during C1 vs TR. This suggests that flavonoid biosynthesis was significantly induced by flooding stress. Similarly, in the C2 vs R comparison, differential accumulation patterns persisted, where flavonoid levels failed to fully revert to baseline after 7-day drainage recovery, demonstrating that flooding-induced damage to quinoa was not entirely alleviated.

### 3.3 Analysis of differential accumulation of flavonoid metabolites

**Figure 2:**
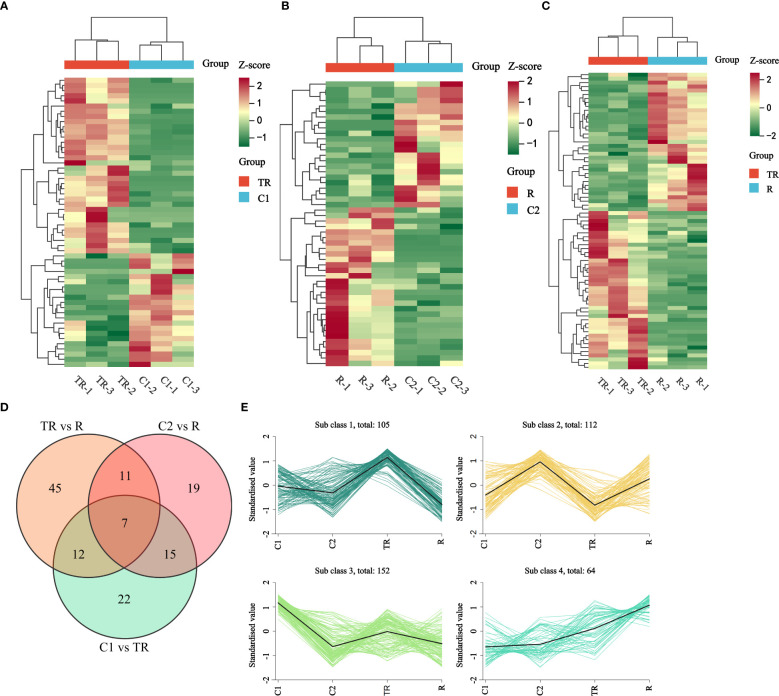
**(A)** Thermogram of differential metabolite accumulation of flavonoids in C1 vs TR. **(B)** Thermogram of differential metabolite accumulation of flavonoids in C2 vs R. **(C)** Thermogram of differential metabolite accumulation of flavonoids in TR vs R. **(D)** Venn diagram of differential accumulation of flavonoid metabolites. **(E)** K-means plot of relative content of differential metabolites of flavonoids. Horizontal coordinates indicate samples and vertical coordinates indicate metabolite abundance after centering and normalization.

In the comparison of C1 vs. TR, 56 differential flavonoid metabolites were identified, with 22 down-regulated and 34 up-regulated (**Figure 2A**; **Supplementary Table 4**). For C2 vs. R, 52 differential flavonoid metabolites were detected, including 23 down-regulated and 29 up-regulated (**Figure 2B**; **Supplementary Table 5**). In the TR vs. R group, 75 differential flavonoid metabolites were observed, of which 40 were down-regulated and 35 up-regulated (**Figure 2C**; **Supplementary Table 6**). The top three metabolites with the largest absolute Log2 Fold Change values across the three comparative groups were summarized in **Table 1**. We observed that the highest-fold-change metabolites in all three comparisons belonged to the flavonols and flavones subclasses. Notably, Isotamarixin was consistently identified across all three comparative groups, suggesting its potential critical regulatory role in quinoa’s response to flooding stress. Furthermore, Galangin-7-O-glucoside, detected in both the C1 vs TR and C2 vs R comparisons, underscores its biologically significant involvement in adaptive mechanisms. In summary, the accumulation of flavonoids was both up-regulated and down-regulated in the three comparison groups of C1 vs TR, C2 vs R, and TR vs R, suggesting that flavonoids can respond to the flooding stress suffered by quinoa during the filling stage through changes in accumulation. Wayne’s analysis of flavonoid differential metabolites (**Figure 2D**; **Supplementary Table 7**) showed 22 flavonoid differential metabolites specific to C1 vs TR, dominated by flavonols (11) and flavones (7), and 19 flavonoid differential metabolites specific to C2 vs R, dominated by Flavonols (5) and flavones (7); the number of flavonoid differential metabolites specific to TR vs R was 45, dominated by flavones (19) and flavonols (14). Seven differential metabolites were co-occurring in the three comparison groups, mainly Flavones (4). This suggests that flavonols and flavones play an important role in the response of quinoa to flooding stress.

**Table 1:** Top three absolute values of Log2 Fold Change in flavonoid differential metabolites.

Comparison group	Compounds	Class II	Log2 Fold Change
C1 vs TR	Gossypetin-3-O-glucoside	Flavonols	14.93
	Galangin-7-O-glucoside	Flavones	13.94
	Isotamarixin	Flavonols	-17.65
C2 vs R	Isotamarixin	Flavonols	16.97
	Pedaliin	Flavones	-16.32
	Galangin-7-O-glucoside	Flavones	-13.86
TR vs R	Isotamarixin	Flavonols	16.97
	Pedaliin	Flavones	-18.01
	Orientin	Flavones	-14.60

The flavonoid differential metabolites were subjected to K-means clustering analysis after standardized and centralized processing. The subclass categorization criteria were defined as follows: the similarity between data objects was measured by inter-sample distance, where smaller distances indicated higher similarity and a higher likelihood of belonging to the same subclass. The results revealed that flavonoids were clustered into four subclasses: Subclass1 (105), Subclass2 (112), Subclass3 (152), and Subclass4 (64) (**Figure 2E**). Subclass2 and Subclass3 likely represent flavonoids that naturally accumulate or decline with developmental progression. Notably, metabolites clustered in Subclass1 exhibited significantly higher levels under flooding stress (TR) compared to the control (C1, C2) and recovery groups (R), suggesting rapid accumulation in response to short-term hypoxia and subsequent sharp decline post-recovery. In Subclass4, metabolite levels in TR and R were higher than in C1 and C2, with R surpassing TR. It is hypothesized that these metabolites may be involved in long-term adaptation to flooding stress and mitigation of oxidative damage during recovery, thus maintaining elevated levels in R. The differential dynamics of these two subclasses highlight a dual “acute-chronic” regulatory strategy in quinoa to combat flooding stress through flavonoid metabolism.

### 3.4 Functional annotation and enrichment analysis of the flavonoid differential metabolite KEGG

The annotation results of KEGG database indicate that the flavonoid metabolites among different treatments were mainly involved in flavonoid biosynthesis (ko00941), phenylpropanoid biosynthesis (ko00940) and flavone and flavonol biosynthesis (ko00944). The differential metabolites annotated in these three pathways are shown in **Table 2**. In C1 vs TR, a total of 74 metabolites were annotated in the three pathways, of which 10 were significantly different; in C2 vs R, a total of 73 metabolites were annotated in the three pathways, of which 13 were significantly different; and in TR vs R, a total of 73 metabolites were annotated in the three pathways, of which 19 were significantly different. The number of differentially different metabolites enriched in these three pathways was relatively similar among the three comparison groups; however, in terms of significantly different metabolites, the TR vs R group was enriched in the highest number, while the C1 vs TR group was enriched in the lowest number.

**Table 2:** Significant changes in KEGG statistics after flooding and restoration treatments.

Class	Kegg pathway	Ko ID	Sig. compound	Compound
C1 vs TR	Flavonoid biosynthesis	ko00941	5	34
	Phenylpropanoid biosynthesis	ko00940	3	19
	Flavone and flavonol biosynthesis	ko00944	2	21
C2 vs R	Flavonoid biosynthesis	ko00941	6	33
	Flavone and flavonol biosynthesis	ko00944	4	21
	Phenylpropanoid biosynthesis	ko00940	3	19
TR vs R	Flavonoid biosynthesis	ko00941	10	33
	Phenylpropanoid biosynthesis	ko00940	5	19
	Flavone and flavonol biosynthesis	ko00944	4	21

Kegg pathway: name of the pathway; Ko ID: ko number of the pathway in the KEGG database; Sig. compound: number of differentially significant metabolites annotated by KEGG in the pathway; Compound, number of metabolites detected that belong to the pathway.

### 3.5 Transcriptome and differentially expressed gene analysis

To intuitively demonstrate the dispersion patterns among the 12 samples, we constructed a principal component analysis (PCA) plot based on gene expression levels (**Supplementary Figure 2A**). The results revealed strong intra-group reproducibility and distinct inter-group separation, with PC1 accounting for 30.48% and cumulative reaching 54.43%, indicating high reliability for subsequent analyses. We identified 552 flavonoid biosynthesis-related genes enriched in three pathways: Flavonoid biosynthesis (ko00941), Phenylpropanoid biosynthesis (ko00940), and Flavone and flavonol biosynthesis (ko00944). These genes’ FPKM expression values were centered, normalized, and subjected to hierarchical clustering analysis, visualized through a clustering heatmap (**Supplementary Figure 2B**). The results demonstrated that the 12 samples clustered into four groups corresponding exactly to the 4 experimental treatments, with clear differentiation in gene expression profiles between treatments.

**Figure 3:**
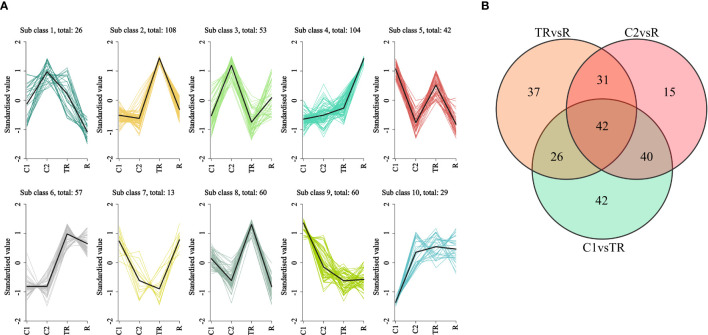
**(A)** K-means clustering plot. Horizontal coordinates indicate samples and vertical coordinates indicate expression after centering and normalization. **(B)** Wayne plots of differential genes in different subgroups.

Genes within the same subclass exhibited similar expression trends across experimental groups, strongly suggesting shared functional roles. K-means clustering analysis was performed on the centralized and standardized FPKM values of 552 flavonoid biosynthesis-related genes (**Figure 3A**). The results categorized these genes into 10 distinct subclasses, with Subclass2 (108) and Subclass 4 (104) containing the largest number of genes. In Subclass 2, gene expression levels surged dramatically under waterlogging stress (TR), significantly exceeding those in other groups (C1, C2, and R), Indicating that these genes were strongly induced by flooding stress. For Subclass 4, gene expression sharply increased during post-flooding recovery (R), surpassing all other groups, suggesting these genes may regulate flavonoid accumulation to mitigate oxidative damage during reoxygenation. Additionally, genes in Subclass 6 displayed markedly higher expression in TR and R groups compared to controls (C1 and C2), implying their persistent regulatory role in countering oxidative stress. Notably, Subclass 7 genes exhibited declining expression from C1 to C2 but rising expression from TR to R. This divergent expression pattern between control and stress conditions strongly suggests their critical involvement in quinoa’s adaptive response to flooding. Differential genes associated with flavonoid synthesis were plotted on a Wayne diagram, and it was found that there were 42 differential genes common to the three comparison groups, and 42, 15, and 37 differential genes specific to C1 vs TR, C2 vs R, and TR vs R, respectively **Figure 3B**).

### 3.6 Analysis of GO and KEGG associated with flavonoid metabolites in the transcriptome

As shown in **Table 3**, all expressed genes in the transcriptome were enriched only in the Molecular Function and Biological Process Ontology entries in the GO entry. In C1 vs TR, C2 vs R, and TR vs R, the differentially expressed genes are all enriched in flavonoid sulfotransferase activity (GO:1990135) in molecular function and flavonoid biosynthetic process (GO:0009813), flavonoid metabolic process (GO:0009812), phenylpropanoid biosynthetic process (GO:0009699), phenylpropanoid catabolic process (GO:0046271), and phenylpropanoid metabolic process (GO:0009698) in biological process. However, there were differences in the number of genes enriched in each group, with C1 vs TR, C2 vs R, and TR vs R enriched for a total of 252, 299, and 347 differential genes in these six pathways, respectively.

**Table 3:** Significant enrichment pathways of flavonoids in GO and KEGG.

	Class	GO or KEGG level 1	GO or KEGG pathway	Description	Count all
GO	C1 vs TR	Biological process	GO:0009813	flavonoid biosynthetic process	58
		Biological process	GO:0009812	flavonoid metabolic process	74
		Molecular function	GO:1990135	flavonoid sulfotransferase activity	7
		Biological process	GO:0009699	phenylpropanoid biosynthetic process	77
		Biological process	GO:0046271	phenylpropanoid catabolic process	5
		Biological process	GO:0009698	phenylpropanoid metabolic process	89
	C2 vs R	Biological process	GO:0009813	flavonoid biosynthetic process	52
		Biological process	GO:0009812	flavonoid metabolic process	61
		Molecular function	GO:1990135	flavonoid sulfotransferase activity	5
		Biological process	GO:0009699	phenylpropanoid biosynthetic process	82
		Biological process	GO:0046271	phenylpropanoid catabolic process	10
		Biological process	GO:0009698	phenylpropanoid metabolic process	89
	TR vs R	Biological process	GO:0009813	flavonoid biosynthetic process	56
		Biological process	GO:0009812	flavonoid metabolic process	69
		Molecular function	GO:1990135	flavonoid sulfotransferase activity	7
		Biological process	GO:0009699	phenylpropanoid biosynthetic process	96
		Biological process	GO:0046271	phenylpropanoid catabolic process	12
		Biological process	GO:0009698	phenylpropanoid metabolic process	107
KEGG	C1 vs TR	Metabolism	ko00944	Flavone and flavonol biosynthesis	28
		Metabolism	ko00941	Flavonoid biosynthesis	64
		Metabolism	ko00400	Phenylalanine, tyrosine and tryptophan biosynthesis	18
		Metabolism	ko00940	Phenylpropanoid biosynthesis	104
	C2vsR	Metabolism	ko00944	Flavone and flavonol biosynthesis	20
		Metabolism	ko00941	Flavonoid biosynthesis	57
		Metabolism	ko00400	Phenylalanine, tyrosine and tryptophan biosynthesis	19
		Metabolism	ko00940	Phenylpropanoid biosynthesis	94
	TR vs R	Metabolism	ko00944	Flavone and flavonol biosynthesis	17
		Metabolism	ko00941	Flavonoid biosynthesis	48
		Metabolism	ko00400	Phenylalanine, tyrosine and tryptophan biosynthesis	16
		Metabolism	ko00940	Phenylpropanoid biosynthesis	107

GO or KEGG level 1: the first level of functional classification in GO or KEGG; GO or KEGG pathway: the GO or ko number of the pathway in the GO or KEGG database; Description: the corresponding functional description of the GO or KEGG pathway; Count all: the number of genes significantly enriched in each pathway.

Statistical analysis was performed to assess the significant enrichment of all expressed genes from the transcriptome in flavonoid biosynthesis-related KEGG pathways (**Table 3**). The results showed that the differential genes in the three comparison groups were mainly enriched in flavone and flavonol biosynthesis (ko00944), flavonoid biosynthesis (ko00941), phenylalanine, tyrosine and tryptophan biosynthesis (ko00400) and phenylpropanoid biosynthesis (ko00940). C1 vs TR, C2 vs R and TR vs R were enriched for a total of 214, 190 and 188 differential genes in the above four pathways.

### 3.7 Joint metabolomic and transcriptomic analysis of flavonoid regulation in quinoa leaves under flooding stress during the filling stage

**Figure 4:**
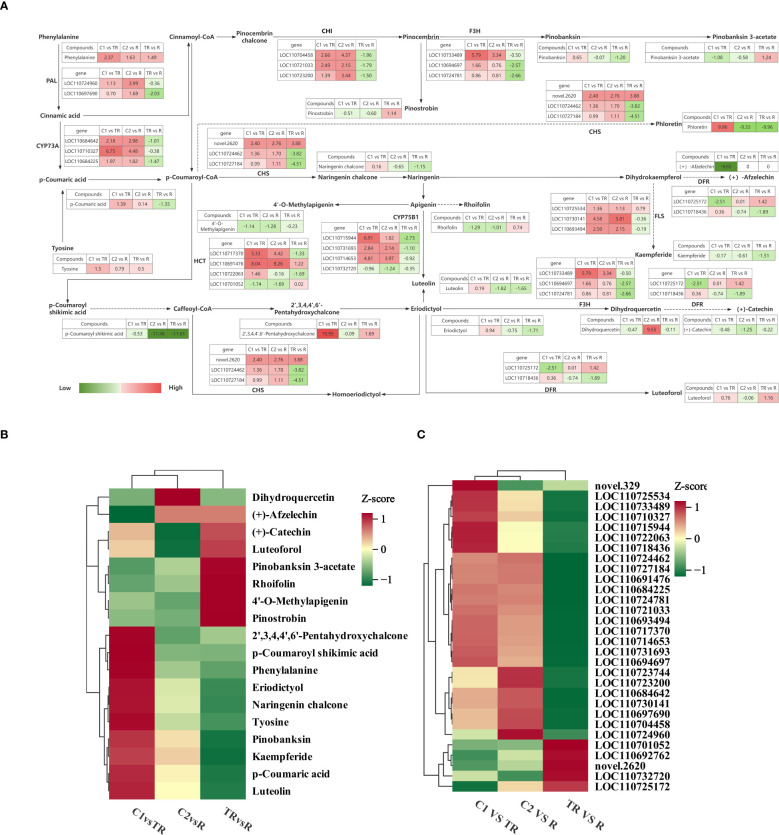
**(A)** Flavonoid response mechanisms in quinoa leaves during the filling stage under flooding-recovery conditions. Red coloration denotes upregulated expression in the latter treatment group of comparative pairs, while green indicates downregulation, with color intensity scaling proportionally to the absolute Log2Fold-Change values. **(B)** Clustering heat map of key metabolites. **(C)** Clustering heat map of key genes.

To understand the differences in flavonoid biosynthesis in quinoa leaves under flooded, restored, and normal control conditions, we integrated metabolomic and transcriptomic data to construct the mechanism of flavonoid biosynthesis in quinoa leaves under flooded and restored treatments during the filling stage (**Figure 4A**). A total of 9 enzymes and 18 metabolites regulated by 30 genes were found to play key roles in response to flooding stress.

Phenylalanine is catalyzed by phenylalanine ammonia-lyase (PAL) [EC: 4.3.1.24] for the synthesis of Cinnamic acid, and two of the genes in PAL are up-regulated for expression in C1 vs TR, C2 vs R, and down-regulated for expression in TR vs R. Cinnamic acid synthesis of p-Coumaric acid was catalyzed by trans-cinnamate 4-monooxygenase (CYP73A) [EC: 1.14.14.91]. Up-regulation of CYP73A expression in C1 vs TR, C2 vs R resulted in up-regulation of p-coumaric acid accumulation, and down-regulation in TR vs R resulted in down-regulation of p-coumaric acid accumulation, suggesting a positive correlation between CYP73A and p-coumaric acid. Synthesis of p-coumaroyl shikimic acid from p-coumaroyl-CoA catalyzed by shikimate o-hydroxycinnamoyl transferase (HCT) [E2.3.1.133]; up-and down-regulation of some of the genes in HCT resulted in down-regulation of p-coumaroyl shikimic acid accumulation in both C1 vs TR, C2 vs R and TR vs R. Flavonoid 3’-monooxygenase (CYP75B1) [EC:1.14.14.82] catalyzed the synthesis of luteolin from naringenin, and luteolin was up-regulated in C1 vs TR and down-regulated in C2 vs R and TR vs R. Chalcone isomerase (CHI) [EC: 5.5.1.6] catalyzes the synthesis of pinocembrin from Pinocembrin chalcone. CHI is up-regulated in C1 vs TR and C2 vs R, and down-regulated in TR vs R. Catalyzed by naringenin 3-dioxygenase (F3H) [EC:1.14.11.9] pinocembrin forms pinobanksin and eriodictyol forms dihydroquercetin. In C1vsTR, F3H was significantly up-regulated, positively correlated with eriodictyol and negatively correlated with dihydroquercetin. In C2 vs R, F3H was down-regulated and negatively correlated with eriodictyol and positively correlated with dihydroquercetin; in TR vs R, F3H was down-regulated and positively correlated with both eriodictyol and dihydroquercetin. This indicates that under flooding stress, genes respond to flooding stress by upregulating F3H, and after recovery treatment, F3H activity gradually decreases. p-coumaroyl-CoA forms phloretin and naringenin chalcone catalyzed by chalcone synthase (CHS) [EC:2.3.1.74]. CHS was positively correlated with phloretin and naringenin chalcone in C1 vs TR; CHS was negatively correlated with phloretin and naringenin chalcone in C2 vs R; in TR vs R, the genes decreased the accumulation of phloretin and naringenin chalcone through a combination of up- and down-regulation. Under the co-catalysis of bifunctional dihydroflavonol 4-reductase (DFR) [EC: 1.1.1.219] and flavanone 4-reductase (DFR) [EC: 1.1.1.234], dihydrokaempferol formed (+)- afzelechin, dihydroquercetin to form (+)-catechin and eriodictyol to form luteoforol. Among them, the accumulation of (+)-afzelechin was significantly down-regulated in C1 vs TR, and there was no significant change in C2 vs R and TR vs R, indicating that the synthesis of (+)-afzelechin was blocked under flooding stress; the accumulation of (+)-catechin was down-regulated in C1 vs TR, C2 vs R and TR vs R; the accumulation of luteoforol was up-regulated in C1 vs TR and TR vs R, and down-regulated in C2 vs R. Dihydrokaempferol was catalyzed by flavonol synthase (FLS) [EC: 1.14.20.6] for the synthesis of kaempferide. FLS up-regulation in C1 vs TR and TR vs R resulted in down-regulation of kaempferide, and in TR vs R, the genes were both up-and down-regulated, which together resulted in a decrease in kaempferide content.

The changes of 30 key genes and 18 key metabolites in C1 vs TR, C2 vs R and TR vs R were plotted as clustered heatmaps (**Figures 4B, C**). The results showed that the expression and abundance of these genes and metabolites varied significantly across the comparison groups and may play an important role under the quinoa flooding-restoration treatment.

### 3.8 Identification and screening of core genes and transcription factors related to flavonoid synthesis

To investigate the overall correlation between flavonoids in the transcriptome and metabolome of quinoa under flooding stress, we performed CCA of differential genes and differential metabolites in the ko00940, ko00941, and ko00944 pathways in C1 and TR (**Supplementary Figures 3A-C**). The results showed that in the ko00940 pathway, l-tyrosine* (mws0250), l-phenyalanine (pme0021), and p-coumaric acid* (pme1439) were highly associated with *LOC110719131*, *LOC110705359*, and *LOC110733538*, respectively, indicating that these three genes are the core genes regulating l-tyrosine* (mws0250), l-phenyalanine (pme0021), and p-coumaric acid* (pme1439). In the ko00941 pathway, 3-o-acetylpinobanksin (mws1074), phloretin (pme1201), and xanthohumol (mwshy0112) have high associations with *novel. 1604*, *LOC110710327*, and *LOC110725172*, respectively. Afzelechin (pme3285) and 3,4,2 ‘, 4’, 6 ‘- pentahydroxychalcone (lmsp004137) have very high associations with *LOC110717594* and *LOC110710327*, respectively. In the ko00944 pathway, rhoifolin (mws0047) and acacetin (mws0051) were highly correlated with *LOC110719441* and *LOC110732722*, respectively. In summary, *LOC110719131*, *LOC110705359*, *LOC110733538*, *novel.1604*, *LOC110725172*, *LOC110717594*, *LOC110710327*, *LOC110719441*, and *LOC110732722* genes have important roles in the synthesis of the above 10 flavonoid compounds.

To reflect the overall impact of transcriptomic and metabolomic data between groups, it is important to reflect the extent to which changes in different variables affect the other grouping, and thus to discover more precisely the key regulatory phenomena in quinoa leaves under flooding stress during grouting period. We selected the differential genes and flavonoid differential metabolites related to flavonoid synthesis in C1 vs TR to establish the Two - Block Orthogonal Partial Least Squares (O2PLS) model and screened out the important variables affecting the other omics by initially determining the variables with higher correlation and weight in different data sets through the loadings plot (**Supplementary Figure 3D**). The results showed that the top five genes with the greatest effect on flavonoid metabolites were *LOC110695540*, *LOC110733538*, *LOC110725836*, *LOC110691988*, and *LOC110684657*. The top five flavonoid metabolites with the greatest effect on gene expression were nobiletin (mwshy0018), tangeretin (mws0055),5-demethylnobiletin;5-hydroxy-6,7,8,3’,4’-pentamethoxyflavone(pmp000113),1,8-dihydroxy-2,6-dimethoxy-5-{[(2s,3r,4s,5s,6r)-3,4,5-trihydroxy-6-(hydroxymethyl) oxan-2-yl]oxy}xanthen-9-one(wbtn004861) and maesopsin* (lcsn003955). These five genes and metabolites are highly likely to be key factors in the response of quinoa leaves to flooding stress.

**Figure 5:**
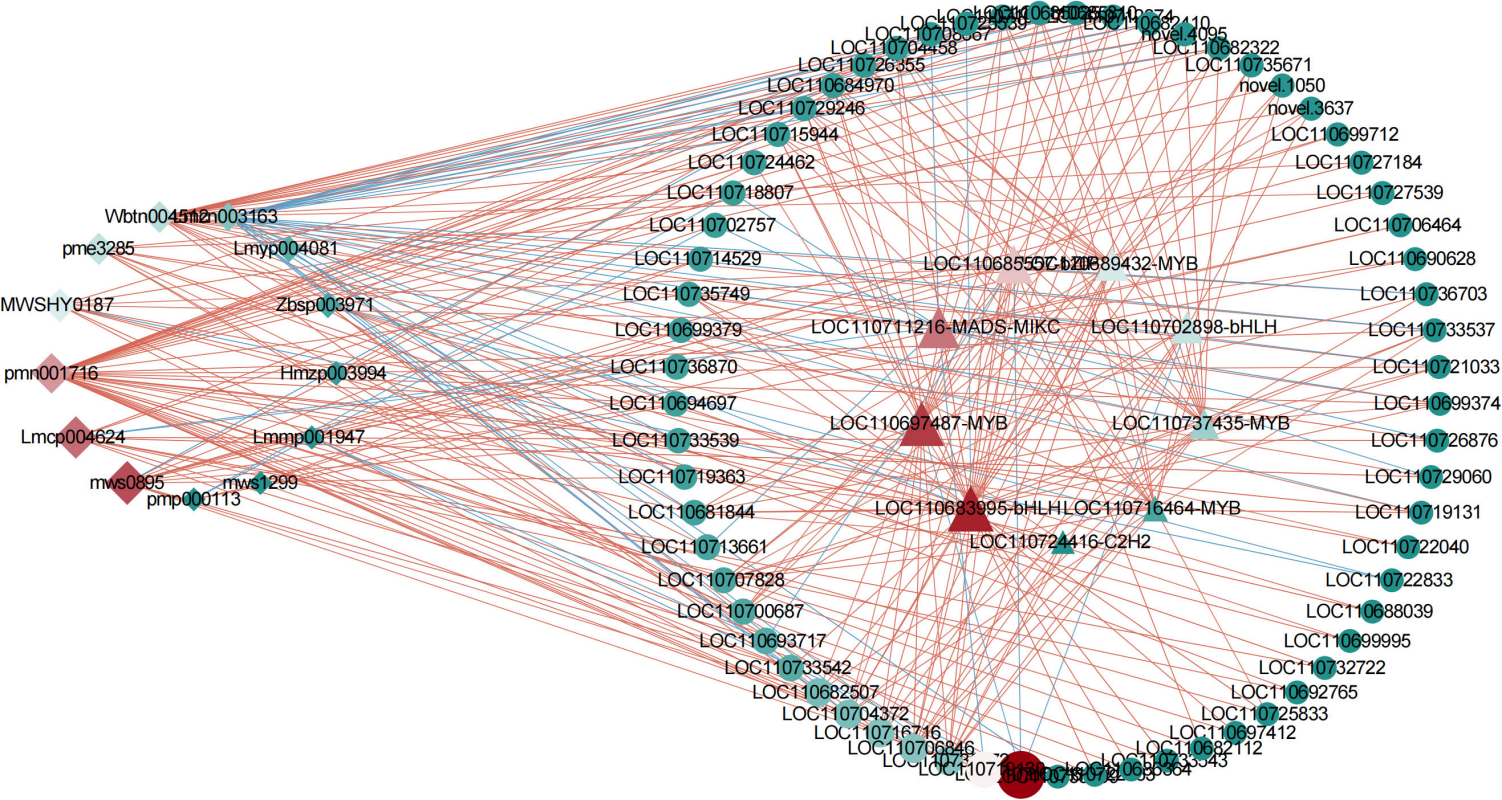
Network interaction diagram. Red lines indicate positive correlation and green is negative correlation. Shapes denote different components: diamond (metabolites), circle (structural genes), triangle (transcription factors). The size of each shape is proportional to the strength of correlation coefficients.

To clarify the effects of transcription factors on flavonoid accumulation under flooding stress, we analyzed the correlation between differential genes and flavonoid differential metabolites in C1 vs TR, C2 vs R, and TR vs R, and selected 50 genes with the top 20 Correlation values and a *P*-value ≤ 0.01 for transcription factor identification, and a total of 10 transcription factors were identified (**Supplementary Table 8**). Subsequently, the structural genes, transcription factors and flavonoid metabolites related to flavonoid synthesis were analyzed for correlation, and the structural genes, transcription factors and flavonoid metabolites with Correlation≥ |0.8| and *P*-value ≤ 0.01 were selected to draw the correlation network diagram (**Figure 5**). The network interactions map showed that the core transcription factors were *LOC110683995* (bHLH), *LOC110697487* (MYB), *LOC110711216* (MADS-MIKC), *LOC110685557* (bZIP) and *LOC110689432* (MYB); the core structural genes were *LOC110715716* and *LOC110719130*; core flavonoid metabolites were Genistin (mws0895), 3-methylkaempferol (lmcp004624), carthamone* (pmn001716) and oroxylin a (mwshy0187), respectively. Two of the transcription factors belonged to the MYB family, and one each to the bZIP, MADS, and bHLH families. RT-qPCR was performed on the five core transcription factors identified in the correlation network diagram to verify their expression. The results indicated that the expression levels were consistent with the RNA-sequencing (RNA-seq) data, thereby confirming the accuracy and reliability of the RNA-seq results (**Supplementary Figure 4**).

## 4 Discussion

Flavonoids have been shown to have the ability to scavenge reactive oxygen species and reduce the production of free radicals and free radicals, and have a positive effect on the removal of harmful substances produced under abiotic stresses (Dong et al., 2020; Li et al., 2021; Liu et al., 2021; Xing et al., 2021). In this experiment, the content of flavonoids in TR treatment was significantly higher than that in C1, and recovery occurred after 7 days of drainage. Perhaps due to the decrease in ROS levels after recovery treatment, the total flavonoid content also decreased accordingly. Studies have demonstrated that transgenic Arabidopsis overexpressing *AcCHI* (chalcone isomerase) exhibited significantly elevated flavonoid content. Compared to wild-type plants, the transgenic lines showed greater biomass, reduced H_2_O_2_ accumulation, and lower relative plasma membrane permeability under salt or osmotic stress conditions, indicating that flavonoids participate in plant abiotic stress responses through ROS scavenging (Feng et al., 2023). In the present study, three CHI genes were upregulated under flooding stress, and total flavonoid content in stressed plants was significantly higher than in the C1 control group. This suggests that the upregulation of CHI genes likely enhances flavonoid biosynthesis, thereby improving ROS scavenging capacity in quinoa. Notably, overexpression of *EbbHLH80* in tobacco increased flavonoid accumulation, resulting in significantly lower ROS levels and enhanced antioxidant enzyme activity compared to wild-type plants (Gao et al., 2023). Therefore, we hypothesize that under flooding stress, flavonoids in quinoa mitigate oxidative damage through dual mechanisms: direct ROS neutralization and indirect upregulation of antioxidant enzyme activity.

Flavonoid metabolites were determined in leaves of quinoa during the filling stage under soil flooding stress using UPLC-MS/MS. A large number of differential metabolites of flavonoids were detected in all treatments, and these differential metabolites were dominated by flavonoids and flavonols. This suggests that flavonoids and flavonols are key elements for quinoa to cope with flooding stress. Meanwhile, the results of SINGHP et al. confirmed that flavonols are effective in increasing plant tolerance to a wide range of abiotic stresses (Singh et al., 2021), which validates the results of the present experiment. Previous studies have demonstrated that Isotamarixin enhances the antioxidant activity of Coreopsis under low-nitrogen conditions (Li et al., 2023). In this study, Isotamarixin consistently ranked among the top three metabolites with the highest fold changes across all three comparative groups, suggesting its potential role in augmenting antioxidant activity and serving as a key mediator in quinoa’s adaptation to flooding stress. Han et al. found that dark-colored quinoa grains contained higher levels of flavonoids than light-colored grains (Han et al., 2019), and Liu et al. found that flavonoids were higher in black and red quinoa than in yellow and white quinoa (Liu et al., 2022b). Quinoa grains are its main edible part, and this study did not investigate the accumulation mechanism of flavonoids in quinoa grains during the filling stage under flooding stress. Subsequent research can fill this gap. Iwashina and Pietta et al. showed that the flavonoid synthesis pathway is involved in stress regulation in plants and that flavonoids can reduce plant damage such as flooding stress, cold stress, and pest feeding, which again validates the role of flavonoids in quinoa under flooding stress in this paper (Pietta, 2000; Iwashina, 2003).

By comparing the differences in flavonoid biosynthesis in quinoa leaves during the filling stage under flooding stress, recovery treatment and normal control conditions, we constructed a map of flavonoid biosynthesis mechanisms. A total of 9 enzymes and 18 metabolites regulated by 30 genes were found to play key roles in response to flooding stress. Studies have shown that chalcone synthase (CHS) has an important influence and key role in plant flavonoid biosynthesis (Anguraj et al., 2018). In citrus, flavonoid biosynthesis is strongly correlated with the expression level of CHS (Wang et al., 2018); in Poncirus trifoliata, the total flavonoid content of leaves is significantly and positively correlated with CHS (Liu et al., 2022c); and, altering the expression level of *EaCHS1* in tobacco affects the accumulation of flavonoids (Lijuan et al., 2015); and, the various expression modes of CHS genes are closely related to the synthesis of different classes of flavonoids (Li et al., 2016). In this experiment, p-coumaroyl-CoA formed phloretin and naringenin chalcone catalyzed by CHS; in C1 vs TR, the expression of CHS showed a positive correlation with the accumulation of naringenin chalcone and phloretin, especially phloretin accumulation was significantly increased, suggesting that CHS plays a key role in the synthesis of phloretin; in TR vs R, down-regulation of some CHS genes led to a decrease in the accumulation of flavonoids in R, suggesting that different CHS genes contribute differently to flavonoid accumulation (Zhou et al., 2013), which is by the results of a previous study. Overexpression of *OsCHI2* (chalcone isomerase 2) from drought-tolerant rice cultivars in drought-sensitive varieties was found to not only regulate flavonoid metabolism but also enhance adaptability to diverse abiotic stresses beyond heat stress (Jayaraman et al., 2021). In the C1 vs TR comparison, three CHI genes were upregulated, whereas all three genes showed downregulation in TR vs R. We hypothesize that these genes positively regulate quinoa’s flooding resistance, which could be further validated through overexpression assays in Arabidopsis thaliana. Furthermore, three FLS (flavonol synthase)-encoding genes exhibited upregulated expression under flooding stress, correlating positively with total flavonoid content. This observation not only highlights the role of FLS genes in flooding adaptation but also suggests that their upregulation likely drives increased flavonoid accumulation. Notably, heterologous overexpression of *DoFLS1*, a flavonol synthase gene isolated from Dendrobium officinale, in Arabidopsis resulted in a 1.24-fold increase in flavonol content (Yu et al., 2021), a finding consistent with our hypothesis. While this study has not yet conducted functional validation of these genes, future investigations targeting their mechanistic roles could address current research gaps. Quercetin is a flavonoid synthesized via the phenylpropane metabolic pathway, which has excellent antioxidant capacity and significantly enhances plant tolerance to a wide range of abiotic stresses (Singh et al., 2021). In this experiment, the accumulation of dihydroquercetin was significantly up-regulated in C2 vs R, whereas the change was not significant in TR vs R. It is hypothesized that soil drainage did not improve plant performance, but instead may have exacerbated oxidative stress and accelerated plant damage (Hossain et al., 2009; Zhang et al., 2023), resulting in the maintenance of a still high accumulation of dihydroquercetin in R. Numerous studies have shown that differential genes and metabolites are enriched in the flavonoid synthesis pathway in plants under flooding stress, which is consistent with the results of this study (Zhang et al., 2017; Hong et al., 2023; Liang et al., 2023; Ren et al., 2017).

Key structural genes for flavonoid synthesis in plants are usually transcriptionally regulated by transcription factors, among which the MYB transcription factor plays an important role. It regulates the expression of enzyme genes during flavonoid biosynthesis and effectively regulates flavonoid accumulation (Payne et al., 2000; Hichri et al., 2011). It was shown that the transcription factor *PpMYB17* activates genes for key enzymes in flavonoid biosynthesis and affects the accumulation of flavonoids in pear fruits (Premathilake et al., 2020). Zhou et al. found that the novel R2R3-MYB transcription factor, *CmMYB012*, can directly regulate the *CmFNS* to inhibit flavonoid biosynthesis (Zhou et al., 2021). Nakatsuka et al. identified two R2R3-MYB transcription factors from Gentian petals, and after overexpression transgenic Arabidopsis and Tobacco both resulted in increased flavonols in seedlings (Nakatsuka et al., 2012). In this study, a total of two MYB core transcription factors were screened using correlation and network interoperability graph analysis, and it is hypothesized that they play important roles in flavonoid synthesis. MYB6 has been reported to activate F3H and FLS1 expression and promote flavonol accumulation in buckwheat and tobacco plants (Yao et al., 2020). This study identified 6 genes encoding F3H and FLS that exhibited upregulated expression under flooding stress. We hypothesize that these genes may be regulated by the MYB transcription factor family. Future studies could perform transcription factor target gene screening assays on the two core MYB transcription factors identified in this experiment to validate their potential regulatory roles over these 6 candidate genes. Additionally, functional validation of these MYB transcription factors will provide critical insights into the molecular mechanisms underlying quinoa’s adaptation to flooding stress. It has been shown that MYB12 can interact with bHLH3 and bHLH33 to play an important role in proanthocyanidin synthesis (Wang et al., 2017). In our study, a key transcription factor of the bHLH family *LOC110683995*(bHLH) with a very high Betweenness value was also screened, and we hypothesized that this transcription factor might interact with the MYB family genes to co-regulate flavonoid metabolite synthesis. A key transcription factor of the MADS-MIKC and bZIP families was also identified through network interaction mapping, and studies have shown that differential expression of the bZIP family and MADS-MIKC family genes affects flavonoid accumulation (Kim et al., 2023; Zhang et al., 2024). These transcription factors may play an important role in the changes of flavonoid content, which is a key factor in response to flooding stress through flavonoids in quinoa during the grouting period, and need to be further verified in the future to see if they are related to flavonoid biosynthesis.

In summary, quinoa leaves synthesize excess reactive oxygen species under flooding stress during the grouting period, causing oxidative damage and cellular destruction and affecting plant growth (Hernandez et al., 2009). In response to the adverse effects of flooding stress on growth and development, quinoa has evolved a complex signaling network to sense environmental changes and initiate defense responses through transcriptional and metabolic regulation, which is manifested by elevating the activity of antioxidant enzymes and increasing the accumulation of cellular osmotic substances as well as flavonoids, thus effectively resisting flooding stress. Reactive oxygen species, as a signaling molecule for plant defense against abiotic stress, binds to stress-responsive transcription factors under stress, prompting the expression of relevant stress-responsive genes, which in turn promotes the synthesis and accumulation of relevant secondary metabolites. Flavonoids, the largest class of phenolic compounds, are important secondary metabolites in plants, capable of inhibiting the production of reactive oxygen species under stress conditions and possessing significant antioxidant functions (Agati et al., 2012). They play a key role in the response of quinoa leaves to flooding stress during the filling stage.

## 5 Conclusion

In this study, we used a combined transcriptome and metabolome analysis to investigate in depth the differences in the mechanism of flavonoid synthesis in leaves of quinoa during the filling stage under flooding and restoration treatments. A total of 433 flavonoid metabolites were detected in the leaves of quinoa during the filling stage under both flooded and drained treatments, and the regulation of their biosynthesis was mainly focused on two biosynthetic pathways, namely flavonoids as well as flavonoids and flavonols. Integrated multi-omics analysis identified multiple genes and metabolites responsive to flooding stress. Five core transcription factors, two core structural genes and four key metabolites were identified by network interactions mapping. This study elucidates the role of flavonoids in quinoa’s response to flooding stress, providing a robust theoretical foundation for breeding flooding-tolerant quinoa varieties. Subsequent studies could employ transgenic and gene editing technologies to validate the functions of these candidate genes under flooding stress.

## Data Availability

The datasets presented in this study can be found in onlinerepositories. This data can be found in the National Center for Biotechnology Information (NCBI) SRA database, with the bioproject accession number PRJNA1039692.

## Supplementary material

Supplementary Figure 1
**(A)** Plot of PCA scores of flavonoids in each group. **(B)** Circle plot of flavonoid metabolite secondary classification occupancy, the length of the column represents the relative content value of the substance. **(C)** Heat map of flavonoid metabolite clustering.

Supplementary Figure 2
**(A)** PCA plot of samples that are clustered based on gene expression levels. **(B)** Clustering heat map of genes related to flavonoid synthesis.

Supplementary Figure 3
**(A)** Ko00940 pathway CCA map. **(B)** Ko00941 pathway CCA map**. (C)** Ko00944 pathway CCA map. **(D)** O2PLS model loading value map. Omic1:transcriptomics,omic2:metabolomics.

Supplementary Figure 4 Real-time fluorescence quantitative PCR plot. The y-axis represents the RT-qPCR relative quantification results (2^-ΔΔCt) and the normalized fold changes (log2(treatment/control)) of transcriptomic FPKM values.

Supplementary Table 1 Specific primer pairs for selected genes.

Supplementary Table 2 Phenotypic changes in quinoa at filling stage under various treatments.

Supplementary Table 3 All flavonoids statistical table.

Supplementary Table 4 Flavonoid differential metabolites in C1 vs TR.

Supplementary Table 5 Flavonoid differential metabolites in C2 vs R.

Supplementary Table 6 Flavonoid differential metabolites in TR vs R.

Supplementary Table 7 Statistical table of Wayne analysis of flavonoids in each group.

Supplementary Table 8 Core transcription factor list.
