# Supplementary figures and images for: Mechanisms of flavonoids in quinoa’s response to flooding stress in grain filling stage
Source: Front Plant Sci. 2025 May 21;16:1565697. doi: 10.3389/fpls.2025.1565697 (PMC12134624; doi:10.3389/fpls.2025.1565697)

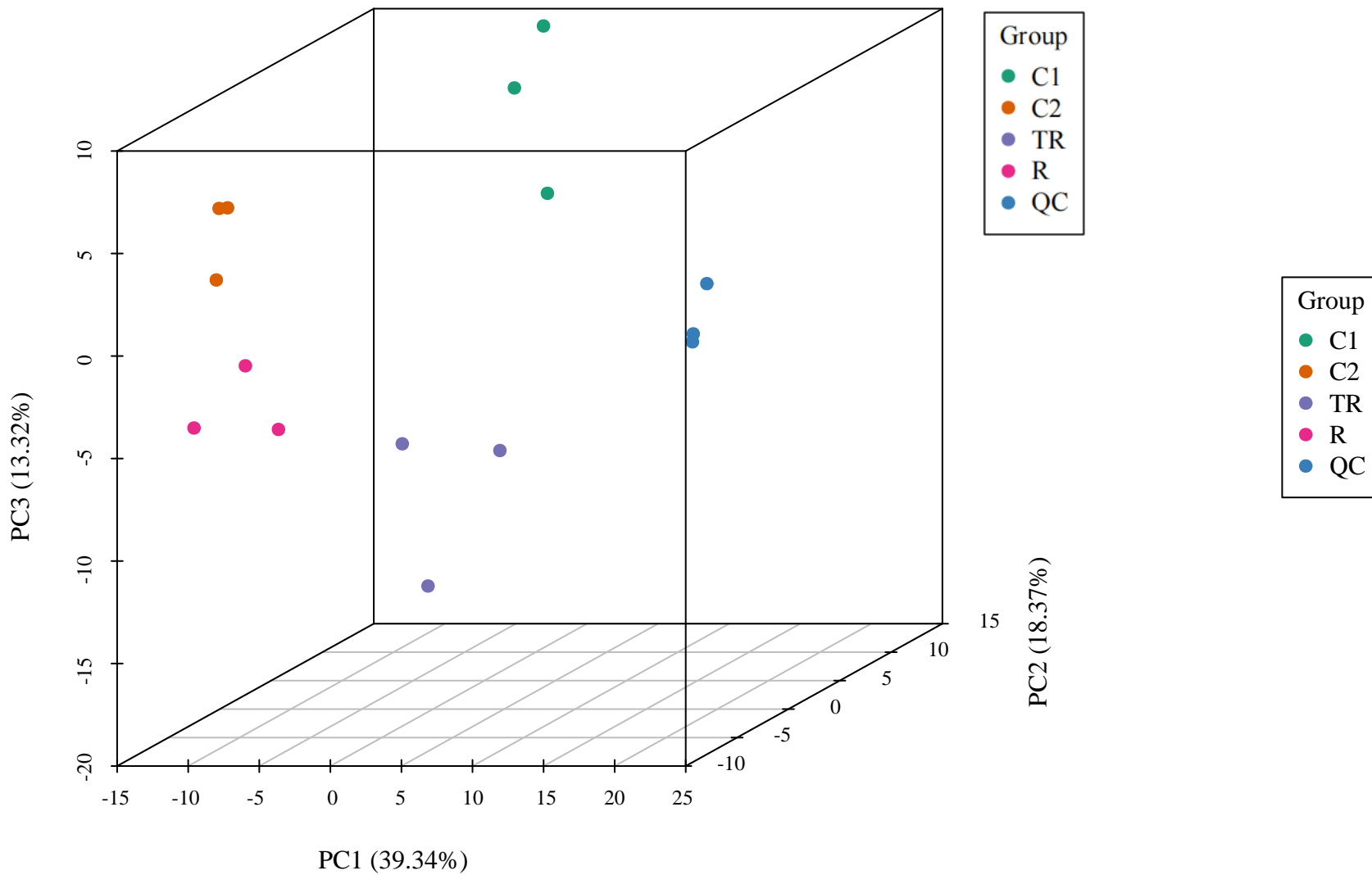

Supplement: Supplementary Figure 1 — (A) Plot of PCA scores of flavonoids in each group. (B) Circle plot of flavonoid metabolite secondary classification occupancy, the length of the column represents the relative content value of the substance. (C) Heat map of flavonoid metabolite clustering. [file DataSheet1.zip › Supplementary materials/Supplementary Figure 1A.pdf]

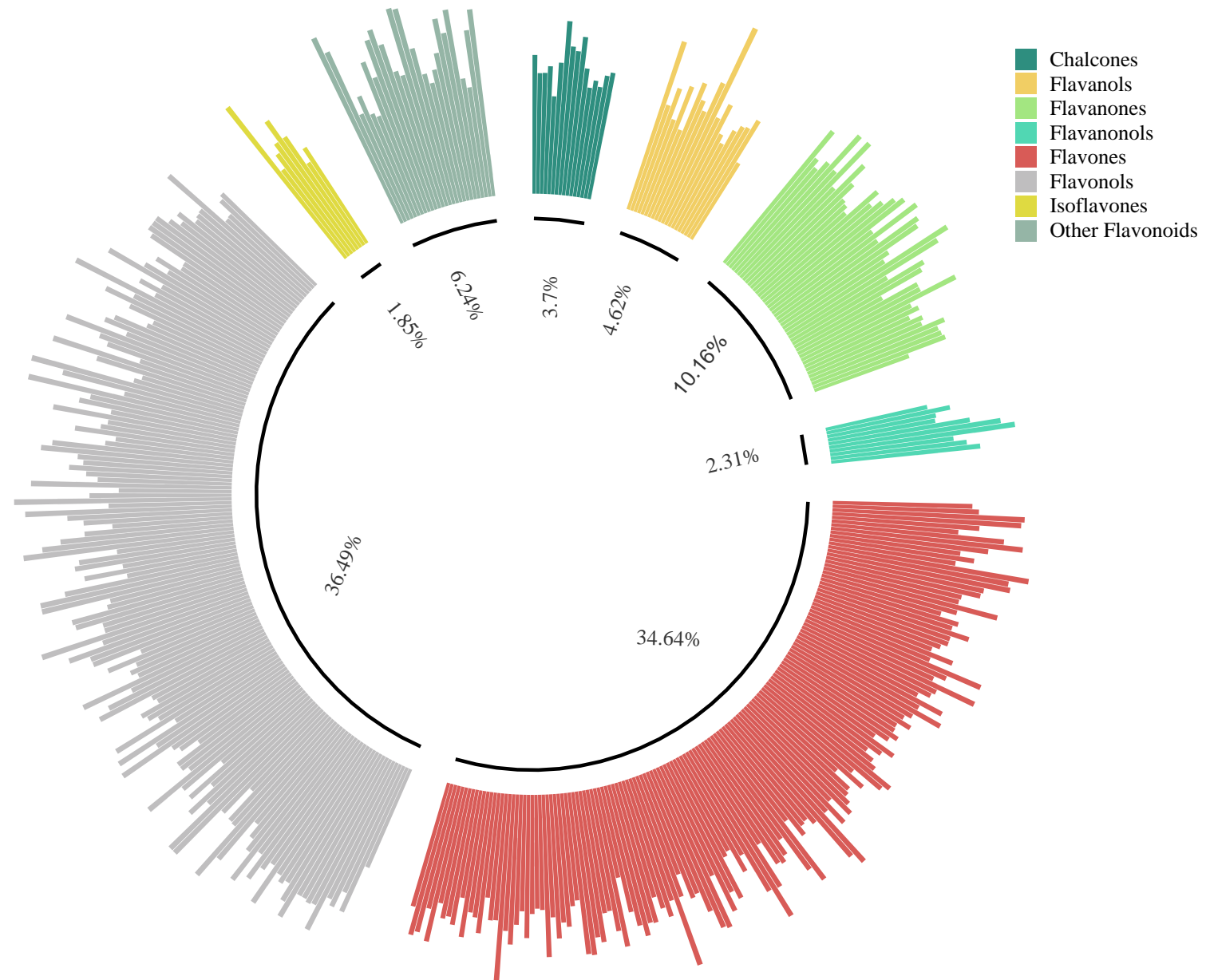

Supplement: Supplementary Figure 1 — (A) Plot of PCA scores of flavonoids in each group. (B) Circle plot of flavonoid metabolite secondary classification occupancy, the length of the column represents the relative content value of the substance. (C) Heat map of flavonoid metabolite clustering. [file DataSheet1.zip › Supplementary materials/Supplementary Figure 1B.pdf]

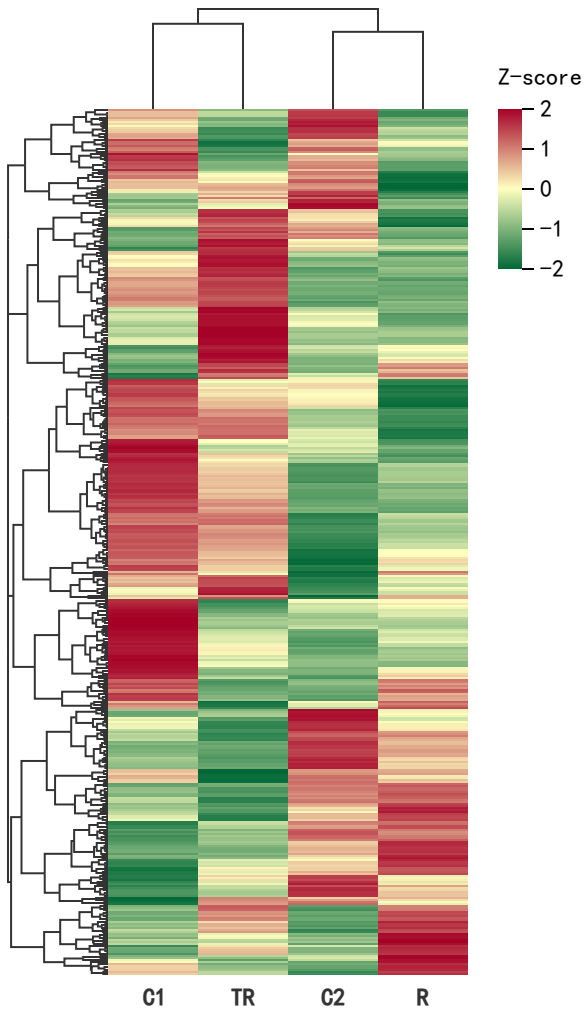

Supplement: Supplementary Figure 1 — (A) Plot of PCA scores of flavonoids in each group. (B) Circle plot of flavonoid metabolite secondary classification occupancy, the length of the column represents the relative content value of the substance. (C) Heat map of flavonoid metabolite clustering. [file DataSheet1.zip › Supplementary materials/Supplementary Figure 1C.pdf]

2D PCA Plot

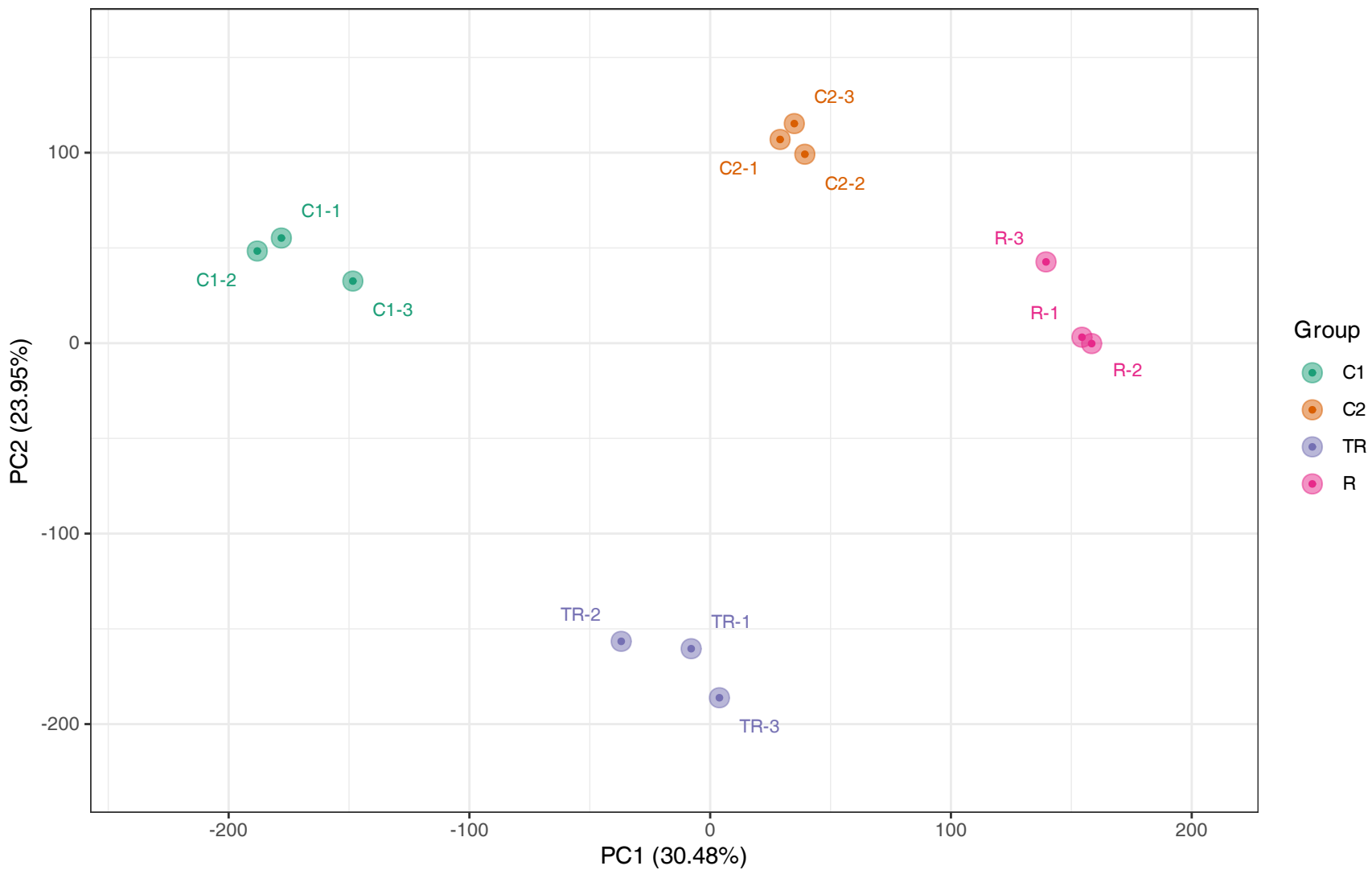

Supplement: Supplementary Figure 1 — (A) Plot of PCA scores of flavonoids in each group. (B) Circle plot of flavonoid metabolite secondary classification occupancy, the length of the column represents the relative content value of the substance. (C) Heat map of flavonoid metabolite clustering. [file DataSheet1.zip › Supplementary materials/Supplementary Figure 2A.pdf]

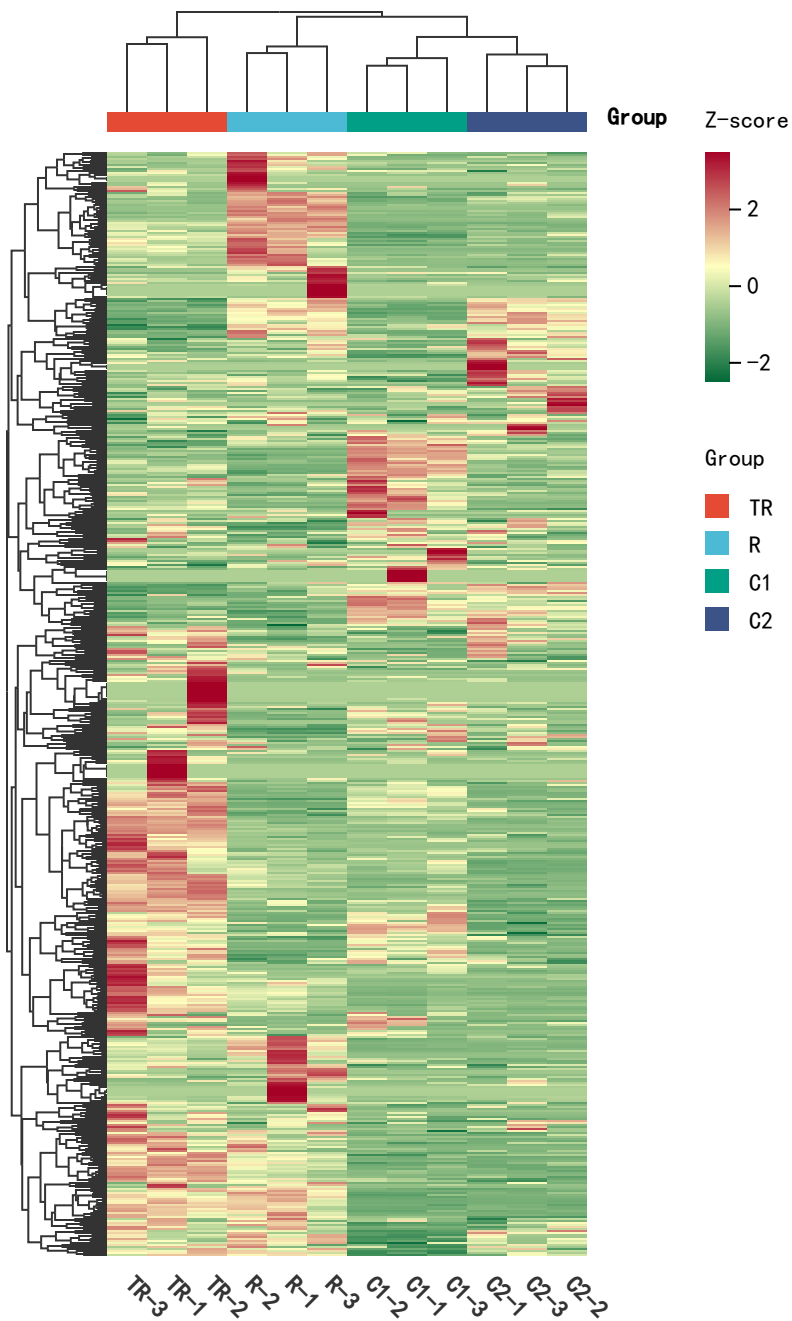

Supplement: Supplementary Figure 1 — (A) Plot of PCA scores of flavonoids in each group. (B) Circle plot of flavonoid metabolite secondary classification occupancy, the length of the column represents the relative content value of the substance. (C) Heat map of flavonoid metabolite clustering. [file DataSheet1.zip › Supplementary materials/Supplementary Figure 2B.pdf]

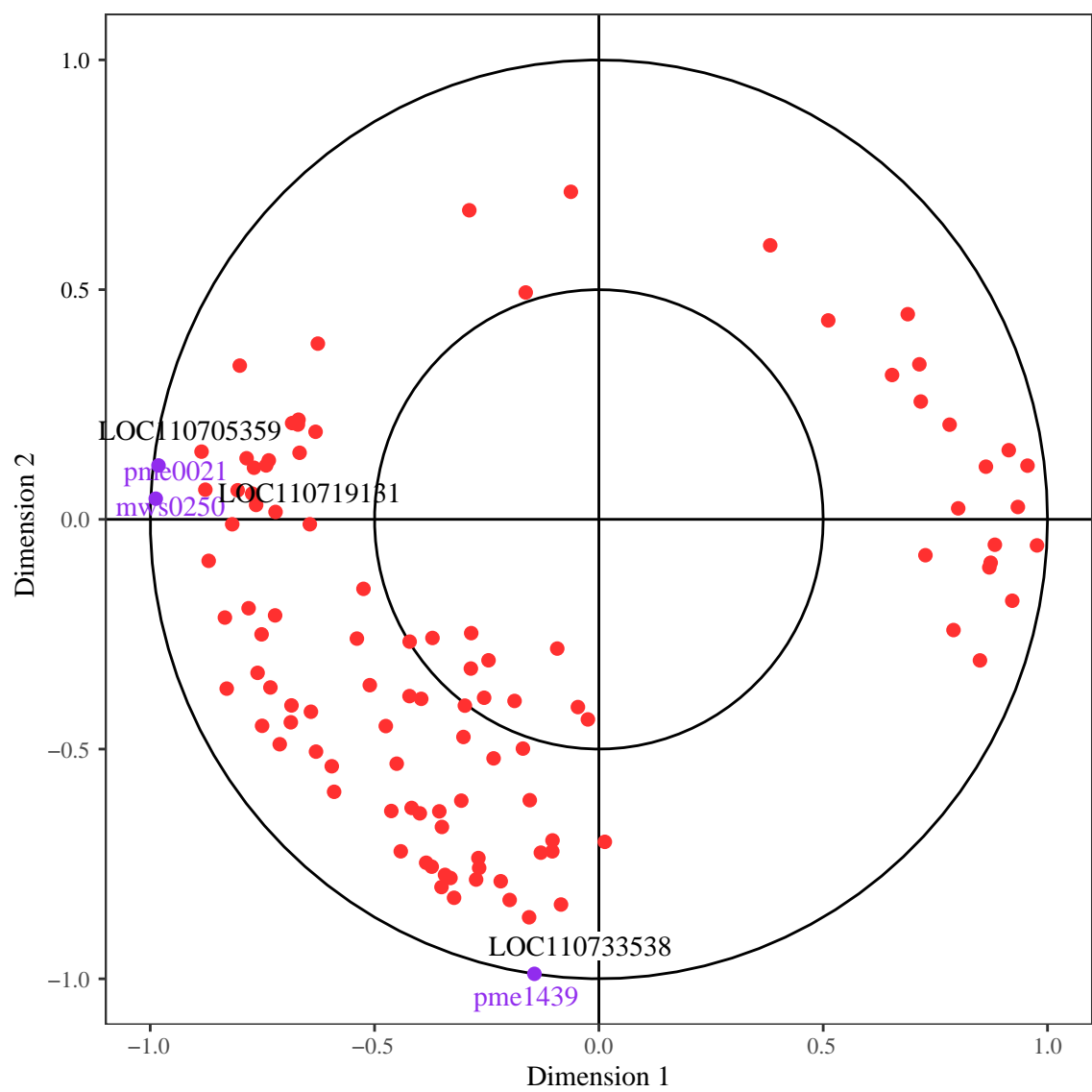

Supplement: Supplementary Figure 1 — (A) Plot of PCA scores of flavonoids in each group. (B) Circle plot of flavonoid metabolite secondary classification occupancy, the length of the column represents the relative content value of the substance. (C) Heat map of flavonoid metabolite clustering. [file DataSheet1.zip › Supplementary materials/Supplementary Figure 3A.pdf]

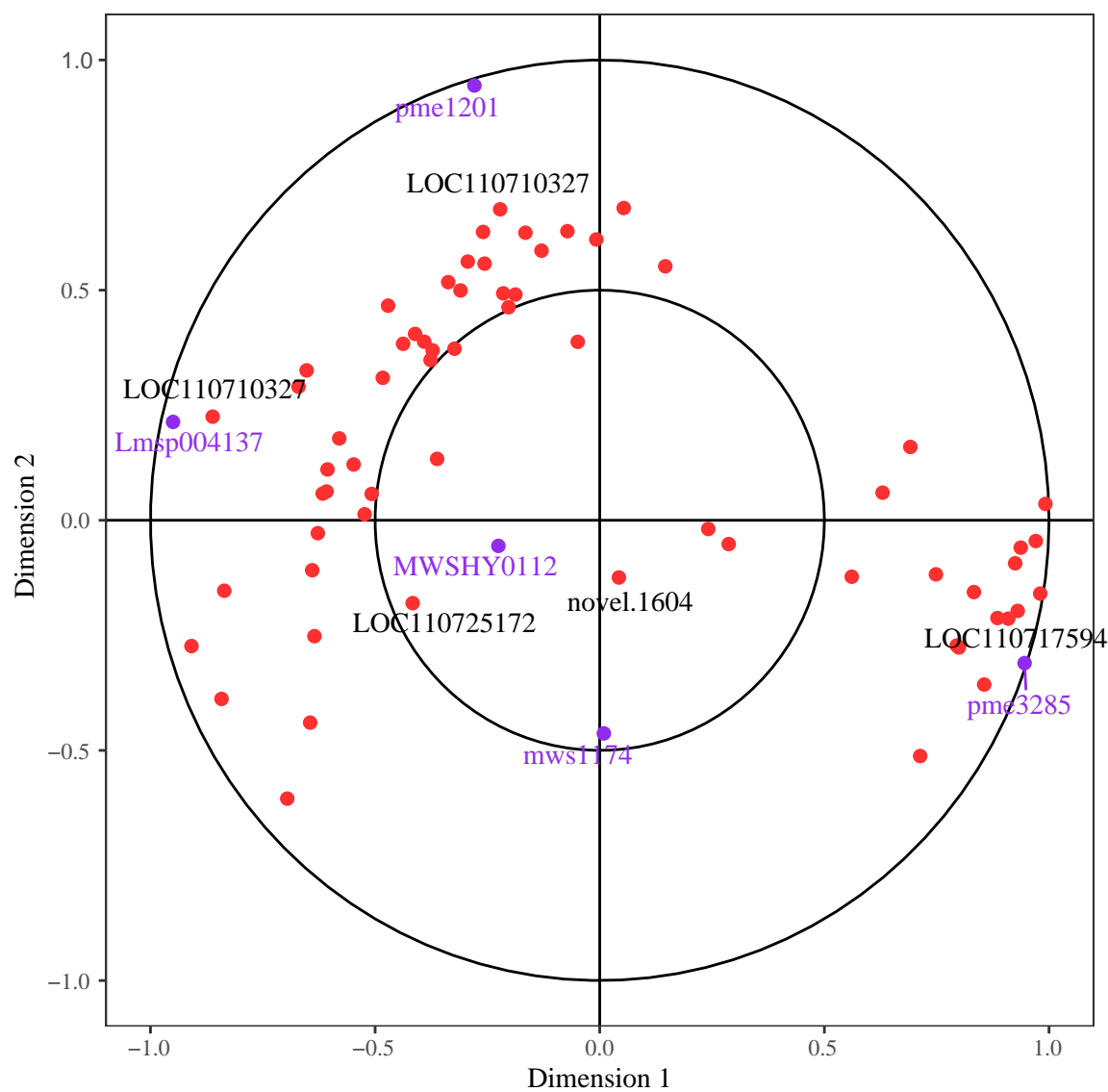

Supplement: Supplementary Figure 1 — (A) Plot of PCA scores of flavonoids in each group. (B) Circle plot of flavonoid metabolite secondary classification occupancy, the length of the column represents the relative content value of the substance. (C) Heat map of flavonoid metabolite clustering. [file DataSheet1.zip › Supplementary materials/Supplementary Figure 3B.pdf]

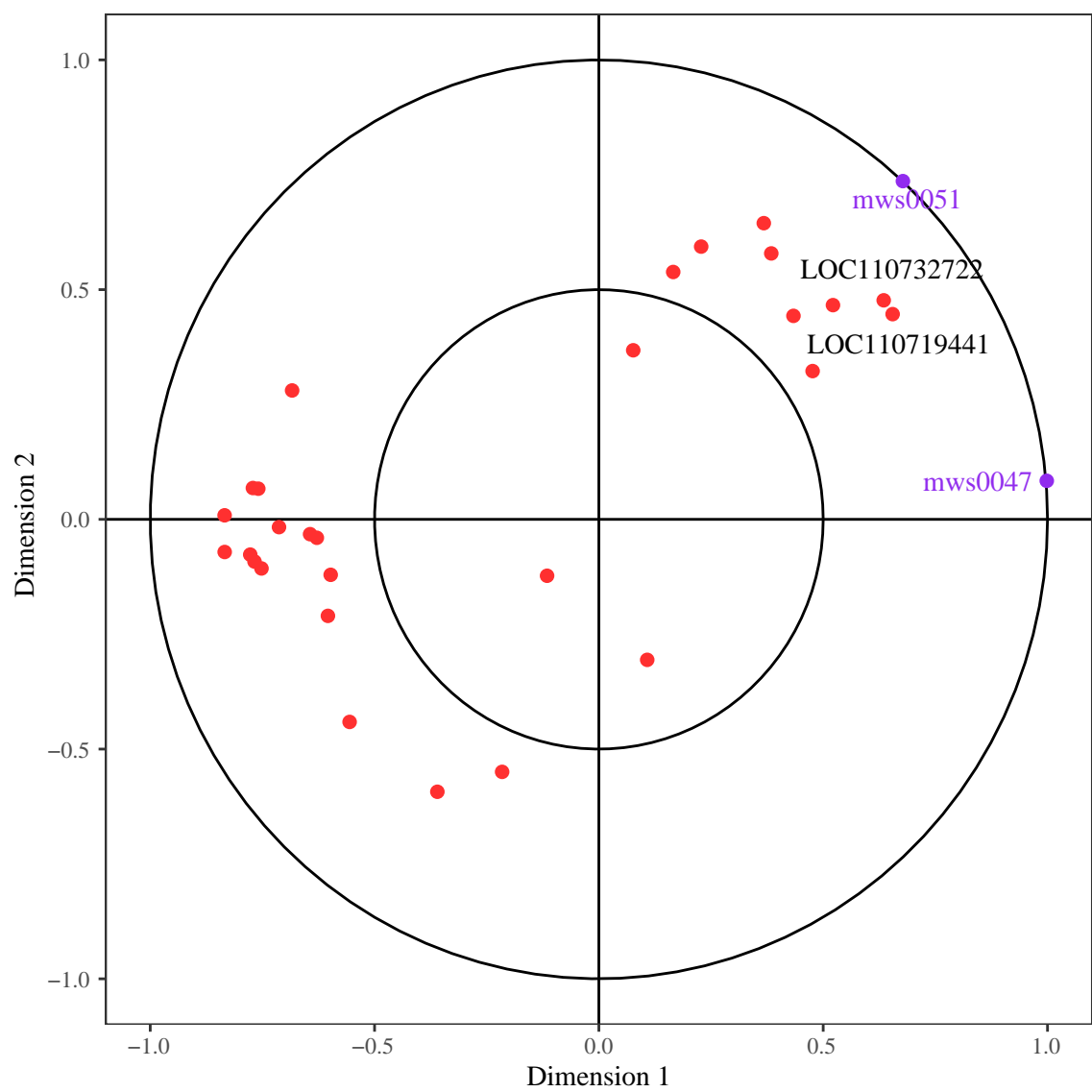

Supplement: Supplementary Figure 1 — (A) Plot of PCA scores of flavonoids in each group. (B) Circle plot of flavonoid metabolite secondary classification occupancy, the length of the column represents the relative content value of the substance. (C) Heat map of flavonoid metabolite clustering. [file DataSheet1.zip › Supplementary materials/Supplementary Figure 3C.pdf]

# Top 20 joint loading

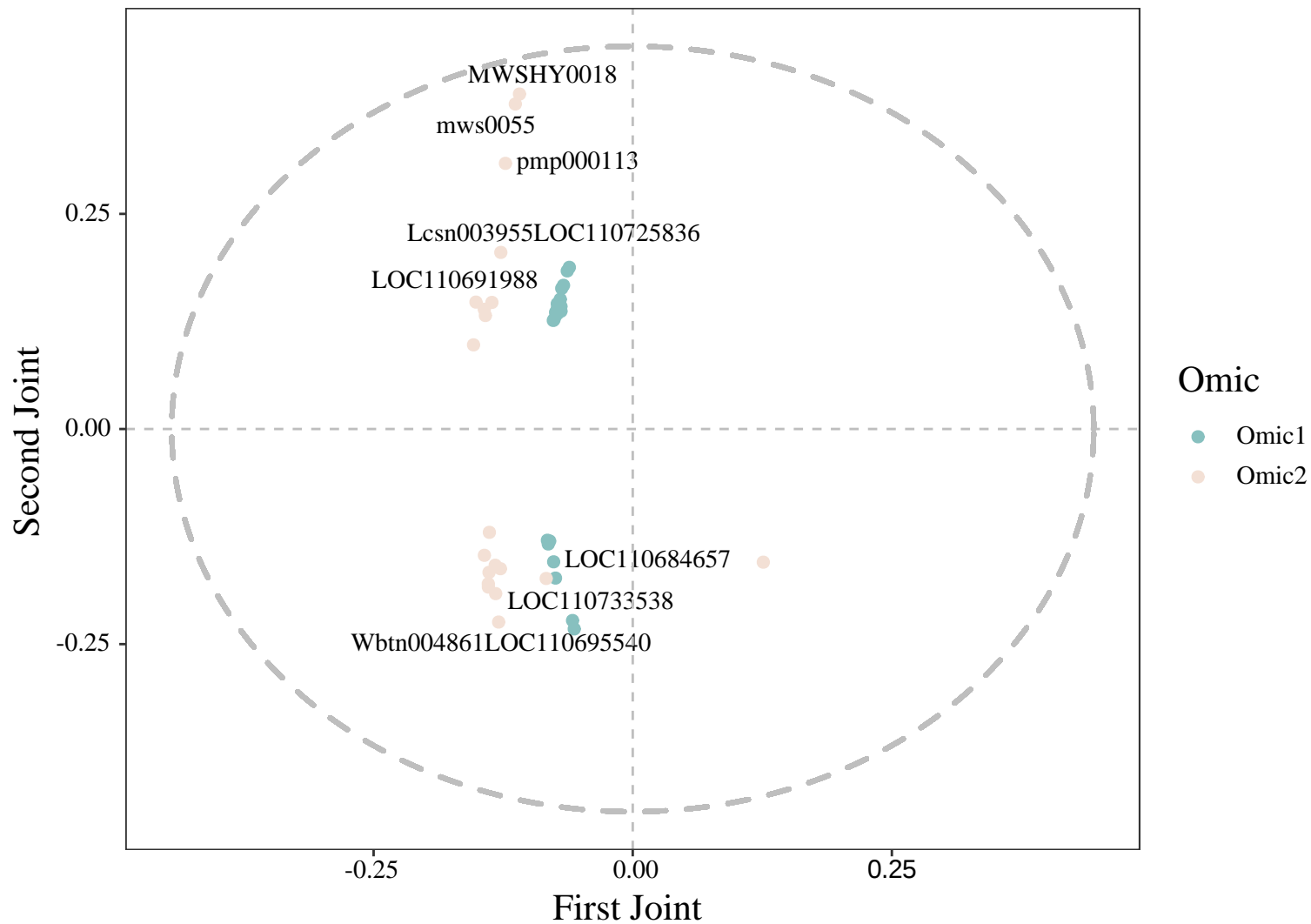

Supplement: Supplementary Figure 1 — (A) Plot of PCA scores of flavonoids in each group. (B) Circle plot of flavonoid metabolite secondary classification occupancy, the length of the column represents the relative content value of the substance. (C) Heat map of flavonoid metabolite clustering. [file DataSheet1.zip › Supplementary materials/Supplementary Figure 3D.pdf]

LOC110685557

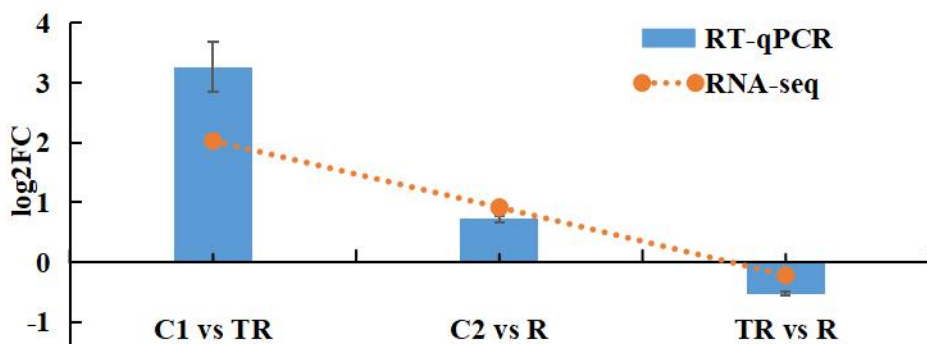

LOC110697487

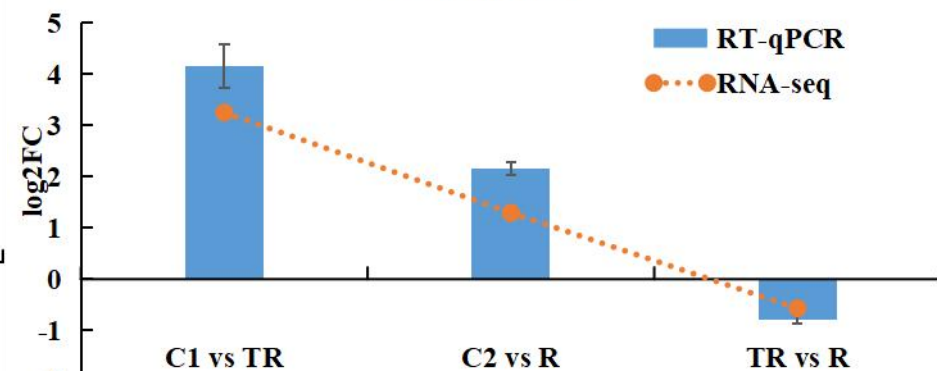

LOC110689432

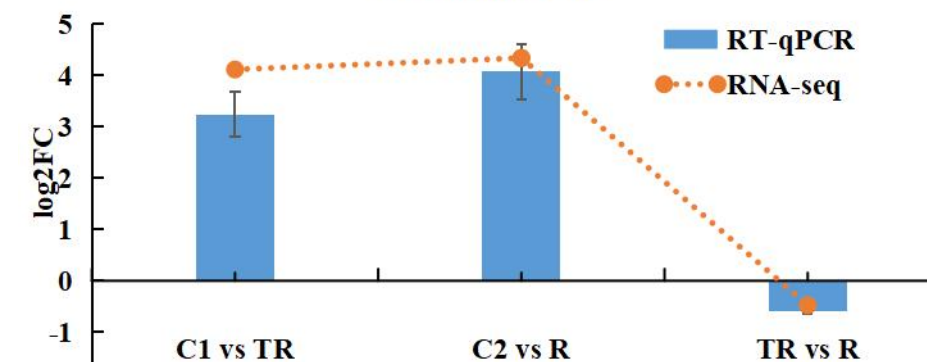

LOC110711216

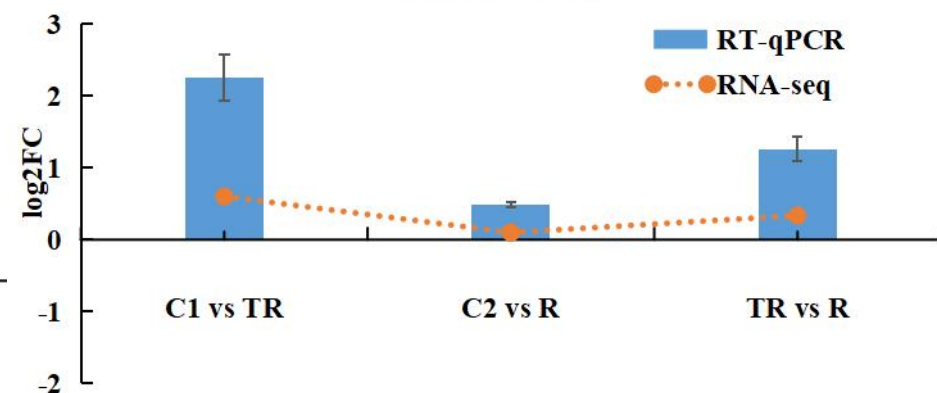

LOC110683995

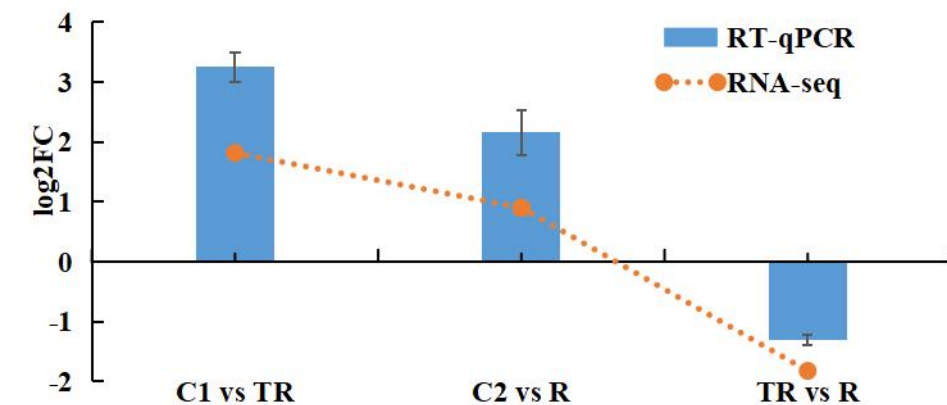

Supplement: Supplementary Figure 1 — (A) Plot of PCA scores of flavonoids in each group. (B) Circle plot of flavonoid metabolite secondary classification occupancy, the length of the column represents the relative content value of the substance. (C) Heat map of flavonoid metabolite clustering. [file DataSheet1.zip › Supplementary materials/Supplementary Figure 4.pdf]
